# Exploring fine-scale urban landscapes using satellite data to predict the distribution of *Aedes* mosquito breeding sites

**DOI:** 10.1186/s12942-024-00378-3

**Published:** 2024-07-07

**Authors:** Claire Teillet, Rodolphe Devillers, Annelise Tran, Thibault Catry, Renaud Marti, Nadine Dessay, Joseph Rwagitinywa, Johana Restrepo, Emmanuel Roux

**Affiliations:** 1 ESPACE-DEV, Univ Montpellier, IRD, Univ Guyane, Univ Reunion, Univ Antilles, Univ Avignon, Maison de la Télédétection, 500 rue Jean‑François Breton, Montpellier Cedex, F‑34093 France; 2 CIRAD, UMR TETIS, Maison de la Télédétection, 500 rue Jean‑François Breton, Montpellier, Cedex, F‑34093 France; 3 TETIS, Univ Montpellier, AgroParisTech, CIRAD, CNRS, INRAE, Maison de la Télédétection, 500 rue Jean‑François Breton, Montpellier, Cedex, F‑34093 France; 4 Direction de la Démoustication, Collectivité Territoriale de Guyane (CTG), 4179 Route de Montabo, Cayenne, Guyane française, 97300 France; 5 International Joint laboratory Sentinela, FIOCRUZ, UnB, IRD, Maison de la Télédétection, 500 rue Jean‑François Breton, Montpellier, Cedex, F‑34093 France

**Keywords:** Remote sensing, *Aedes aegypti*, Urban areas, Prediction, Landscape ecology, Vector control, Arbovirus diseases

## Abstract

**Background:**

The spread of mosquito-transmitted diseases such as dengue is a major public health issue worldwide. The *Aedes aegypti* mosquito, a primary vector for dengue, thrives in urban environments and breeds mainly in artificial or natural water containers. While the relationship between urban landscapes and potential breeding sites remains poorly understood, such a knowledge could help mitigate the risks associated with these diseases. This study aimed to analyze the relationships between urban landscape characteristics and potential breeding site abundance and type in cities of French Guiana (South America), and to evaluate the potential of such variables to be used in predictive models.

**Methods:**

We use Multifactorial Analysis to explore the relationship between urban landscape characteristics derived from very high resolution satellite imagery, and potential breeding sites recorded from *in-situ* surveys. We then applied Random Forest models with different sets of urban variables to predict the number of potential breeding sites where entomological data are not available.

**Results:**

Landscape analyses applied to satellite images showed that urban types can be clearly identified using texture indices. The Multiple Factor Analysis helped identify variables related to the distribution of potential breeding sites, such as buildings class area, landscape shape index, building number, and the first component of texture indices. Models predicting the number of potential breeding sites using the entire dataset provided an R² of 0.90, possibly influenced by overfitting, but allowing the prediction over all the study sites. Predictions of potential breeding sites varied highly depending on their type, with better results on breeding sites types commonly found in urban landscapes, such as containers of less than 200 L, large volumes and barrels. The study also outlined the limitation offered by the entomological data, whose sampling was not specifically designed for this study. Model outputs could be used as input to a mosquito dynamics model when no accurate field data are available.

**Conclusion:**

This study offers a first use of routinely collected data on potential breeding sites in a research study. It highlights the potential benefits of including satellite-based characterizations of the urban environment to improve vector control strategies.

**Supplementary Information:**

The online version contains supplementary material available at 10.1186/s12942-024-00378-3.

## Introduction

According to the World Health Organization, mosquito-transmitted diseases, such as dengue, chikungunya, and Zika, have intensified during the past decades and are responsible for over 700,000 deaths each year. One of its main vectors, *Aedes aegypti*, is strongly adaptable to urban environments and thrives in urban settlements [1], favoring human dwellings and feeding almost exclusively on human blood [2]. Adult females get infected by feeding on the blood from an infected human host and can in turn transmit the disease to another host after pathogen proliferation in the vector (extrinsic incubation period) [3]. After a blood meal, females lay eggs in containers of stagnant water and larvae develop in the water before becoming adult mosquitoes. *Ae. aegypti* breeds mainly in small artificial or natural water containers, such as water storage containers, used tires, plastic containers, clogged gutters, and ornamental plants [4]. The configuration of the urban landscape is known to impact the availability and distribution of such containers, and therefore of potential *Ae. aegypti* breeding sites [5–7].

If the risk of introducing *Ae. albopictus* remains high in French Guiana, *Ae. aegypti* is currently the sole arboviruses vector for dengue, chikungunya, and zika viruses [8]. *Ae. aegypti* is present in nearly all inhabited regions, including smaller human settlements, and even in some wild areas [8]. The abundance of adult mosquitoes is strongly associated with building densities and is therefore higher in densely populated urban areas, in particular but not exclusively in the main cities, such as Saint-Laurent du Maroni, Kourou, Cayenne, Remire Montjoly, and Matoury [9]. In French Guiana, vector control mainly relies on indoor and outdoor insecticide spraying where human cases of any *Aedes*-borne disease have been identified, to control adult mosquitoes, and on routine mechanical or chemical elimination of larval breeding sites in urban areas (see paragraph *2.2 Entomological Data* and [10]). Since the 1940s, the use of various insecticides for vector control in French Guiana has led to the development of resistance in *Ae. aegypti* populations, resulting in reduced efficacy in the territory [10, 11]. Vector control strategies are therefore exploring alternative methods that could target adult mosquitoes, acknowledging the need for more precise larval control in time and space [11]. Several epidemics linked to arboviruses have occurred in recent decades (i.e., 2006, 2009–2010, 2013, and 2020–2021). Since April 2023, French Guiana has been experiencing new dengue fever epidemics, mainly due to serotypes DEN-2 and DEN-3 [12]. Unlike other Latin American countries (Brazil in particular), there are not many studies on dengue fever or its associated vector in French Guiana [13].

Reducing the risks associated with *Aedes*-borne diseases can be achieved by a better understanding of the influence of urban environment factors on the distribution of *Ae. aegypti* breeding sites. If such a knowledge can be achieved by *in-situ* entomological data collection, especially data on potential breeding sites, such a task can be very time-consuming and challenging, given the complexities of obtaining exhaustive data in complex urban environments and is not done in many places.

Remote sensing proved its ability to help characterize urban environmental and climatic variables associated with the mosquito life cycle, including breeding sites [14, 15]. Such an approach can hence provide an effective method to characterize urban landscapes, offering cost-effective and reproducible approaches that can be used at different spatial and temporal resolutions [14, 16]. A wide range of information contributing to the urban landscape characterization can be derived from remote sensing data, such as: land use or land cover [17, 18] and the landscape metrics that can be calculated from it [19, 20], spectral, thermal and texture indices [21–24], and elevation models [25].

Most studies using remote sensing data for modeling vector populations used entomological data associated with presence and/or abundance of adult mosquitoes (BG-Sentinel trap) or larvae (Ovitraps) [26–28]. Another approach consists of using remote sensing data to predict the distribution of potential mosquito breeding sites (i.e., recipients with larvae, with water but without larvae, or without water). Studies employing such approaches are rare, primarily due to the time and resources required for identifying and counting domestic and peridomestic containers by vector control efforts [29–33].

In these studies, input data and variables are usually satellite-derived land surface temperature, land use or land cover (LULC) classifications with few broad classes, in addition to radiometric indices like the normalized difference vegetation index (NDVI). Those are commonly used as predictors in models predicting the presence of* Aedes *breeding sites. However, urban features at fine spatial resolution have rarely been analyzed to inform those models. Arduino et al. [33] studied the impact of micro-environments on positive breeding sites (positive to larvae) using very high resolution Ikonos satellite images, without considering the urban configuration in detail. Different approaches used Haralick texture indices [28] and landscape metrics (Shannon diversity index [34]) for urban space zoning, but not as direct predictors of the number and distribution of breeding sites. As far as we know, urban landscape characteristics at a fine spatial scale have rarely been considered as input variables in mosquito-borne disease models [35], especially when modeling potential breeding sites of *Aedes* mosquitoes.

Various methodological approaches have been used for modeling the distribution of *Aedes* mosquitoes and their breeding sites, such as linear regression [29], species distribution models [30] and more recently Boosted Regression Tree [31]. Machine learning models, such as Random Forest (RF), have already shown good results to study the relationship between ovitraps or adult mosquitoes data and environmental factors [26, 36–38]. However, RF models has never been applied to routinely acquired potential breeding sites data to investigate their complex relationships with urban landscapes.

Hence, using descriptive landscape variables such as texture, landscape metrics, buildings and vegetation heights, this paper proposes an original approach that aims to enhance the identification of relationships between urban landscapes and potential *Ae. aegypti* breeding sites at a fine spatial resolution. Our study analyzes the relationships between urban landscape variables, especially those derived from very high resolution (VHR) satellite imagery, and *in-situ* data on potential breeding sites in cities of French Guiana (South America), while evaluating the capability of these variables to be used in predictive models.

## Materials and methods

### Geographical context

**Fig. 1:**
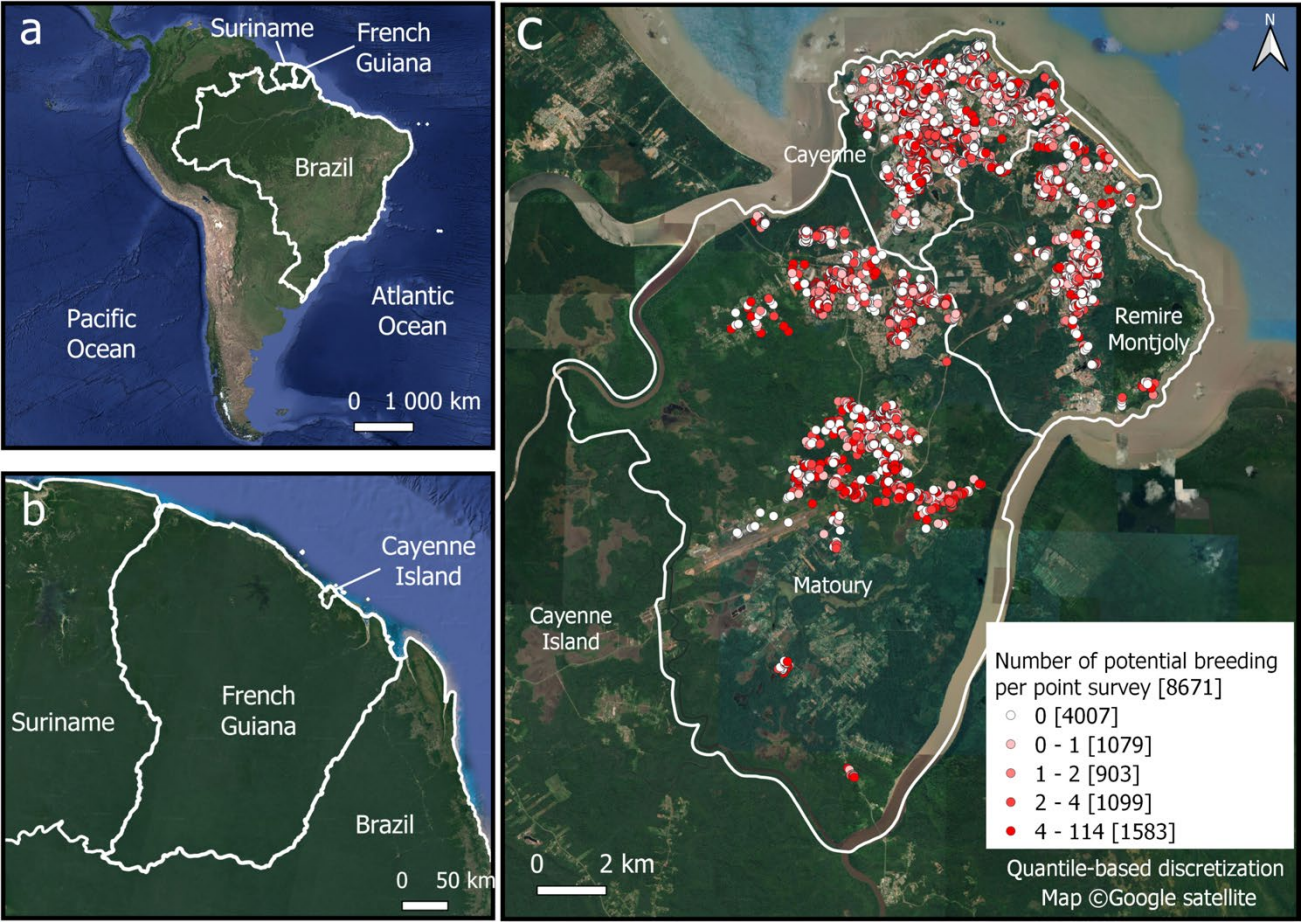
Study Site: (**a**) French Guiana located in South America; (**b**) French Guiana, neighboring countries and Cayenne Island; (**c**) Number of potential breeding sites by prospected location during 2022. The three municipalities of the Cayenne Island (Cayenne, Rémire-Montjoly and Matoury) are delimited with solid white lines

The study area is the Cayenne Island, the largest urban region of French Guiana, a French overseas territory of 83,800 km^2^ and 285,000 inhabitants (2020) located in the northeast of South America, sharing borders with Brazil and Suriname (Fig. 1a, b). ‘Cayenne Island’ is the name given to the peninsula surrounded by the estuaries of the Cayenne River to its west, the Mahury River to its east, and the Atlantic Ocean on its northeast. The peninsula is composed of the cities of Cayenne, Matoury, and Remire-Montjoly covering only 0.25% (i.e. 206.91 km²) of the French Guiana territory but hosting nearly half of the French Guiana population (126,223 inhabitants in 2020). French Guiana is covered by 95% of forest, and has an equatorial climate, characterized by year-round high temperatures and high humidity with an annual mean temperature of 26 °C, an annual mean humidity of 80–90% and between 2000 and 4000 mm of annual precipitation. The year is divided into four seasons: a long rainy season from early April to mid-July, a lengthy dry season from mid-July to mid-November, a short rainy season from mid-November to mid-February, and a brief dry season from mid-February to early April called “little summer of March”. French Guiana regularly faces dengue outbreaks [8], with the latest one that lasted almost a year and a half, from January 2020 to June 2021. During this last epidemic event, nearly 10,900 clinically evocative cases seen in consultations were estimated, and 6195 probable or confirmed cases were identified.

### Entomological data

Various efforts are coordinated by government agencies to prevent, monitor and address dengue outbreaks, including regular field surveys aiming to identify and characterize mosquito breeding sites. A dataset of *Ae. aegypti* breeding sites in French Guiana for 2022 was obtained from the *‘Direction de la Démoustication et des Actions Sanitaires*’ (Direction of mosquito control and health actions) of the ‘*Collectivité Territoriale de Guyane*’ (regional administration - CTG). Each month, randomly selected addresses are inspected for the presence or absence of *Ae. aegypti* larvae. Containers that store water with larvae are recorded as being positive breeding sites, while negative breeding sites are containers with water but no larvae, or without water during the time of prospection but that could have water in the future. This survey is carried out throughout French Guiana, with the territory divided into geographical sectors, subdivided into ‘blocks’ used to plan the monthly field surveys. Depending on the size of the blocks, the number of locations to be visited each month is determined. Only locations visited by agents with geographical coordinates located in Cayenne Island were included in the final dataset. If a container is positive for larvae during a survey, mechanical control or biological treatment are applied and the control action is registered. To characterize the routine visits carried out by vector-control agents, a representation of the number of locations prospected per block is provided in Fig. 3a.

The dataset included 56,985 records acquired from January 4, 2022 to December 30, 2022, 18,576 being located on the island of Cayenne, with 8775 locations actually visited by the agents. Indeed, 52.76% of the sites located on the Cayenne Island could not be inspected due to the absence of inhabitants or a refusal from the owners to conduct the survey. Then, 104 records, corresponding to tire sales zones, garages, and cemeteries, were excluded from the analyses as these places: (i) are known to structurally produce a very high quantity of potential breeding sites and undergo higher sampling efforts, introducing biases in the models; (ii) are not associated with specific urban landscape types, introducing random noise in the modeling step. The effects of such biases and random noise have been revealed by preliminary models that were tested on the entire dataset. As a result, 8.671 records were considered in the study. The number of potential breeding sites per location was obtained by adding positive and negative breeding sites (Fig. 1c). Breeding sites are classified by the CTG into one of 19 types: containers over 200 L, containers under 200 L, small waste, large waste, tires, barrels, building materials, large volumes, manholes, gutters, troughs, green plants, other plants, wells, pits, watercraft, protection, canals, and others. These types were then grouped into larger categories based on their similarity and type of management based on experts’ knowledge (Table 1). Pre-processing of entomological data was carried out with the QGIS software (v. 3.16) [39].

**Table 1:** Types and categories of breeding sites

Categories	Types	Comments	Management
Containers	containers over 200 L	containers with a volume of more than 200 L	emptying / covering / insecticide treatment
	containers under 200 L	containers with a volume of less than 200 L containers with a capacity smaller than barrels	
	barrels	barrels of 200 L	
	well		
Plants	pots plants		regular maintenance of pots and garden
	other plants	ground plants	
Waste	building materials		storage/removal maintenance
	tires		
	watercraft		
	small waste	can be removed manually	
	big waste	can not be removed manually	
	protection	tarpaulin, plastic, fabric… used to cover an object	
Water Management Infrastructure	gutters		maintenance of water management infrastructures
	canals		
	manholes		
	troughs		
	pit		
Large Volume	large volumes	pool, large water basin	emptying / covering
Others	others	unclassified types	

### Satellite-derived and geospatial data

#### Satellite imagery

Pléiades satellite images of the study area were acquired on July 20, 2022. Pléiades images consist of a panchromatic band (0.5 m resampled spatial resolution) and 4 spectral bands (blue, green, red, and near-infrared bands at 2 m spatial resolution). The very high spatial resolution of Pléiades images allows for the observation of fine details within the defined area, including urban vegetation and building arrangement.

#### Textural indices

In this paper, image texture was used to help characterize the arrangement of the urban elements and therefore different urban types (e.g., dense housing, commercial areas, urban parks) [40]. The unsupervised FOTOTEX algorithm [41] has been applied to the Pléaides imagery panchromatic band with a 201-pixel (100.5 m) window size using a block method (each window is processed sequentially block by block in the image, see [42] for more details), generating textural indices indicative of different urban patterns, depending on the distribution and configuration of urban elements. The window size was considered large enough to include several repetitions of patterns of interest and therefore, characterize different types of urban areas. The FOTOTEX algorithm [41] consists in applying a Fourier Transform (FT) to each window, then applying a principal component analysis (PCA), with the windows as statistical individuals, and the spatial frequencies from FT as variables. The first three components of the PCA were used as textural indices. This algorithm has been used in vegetation contexts [42–46], and has recently been applied to identify urban areas [41]. However, this algorithm has never been used in combination with breeding site data and our hypothesis is that such an approach could enhance our knowledge of the links between urban landscape and breeding site distribution, and therefore facilitate the remote sensing-based prediction of breeding site distribution. In this study, the FOTOTEX algorithm was performed using the python package “*fototex 1.5.9*” [41].

#### Spectral indices

Normalized Difference Vegetation Index (NDVI) and Normalized Difference Water Index (NDWI) were derived from the Pléaides images. The NDVI is calculated using the equation $NDVI = NIR - RED / NIR + RED$ where RED and NIR correspond to the reflectance measure in Bands 3 and 4, respectively [47]. The NDVI is widely used for identifying vegetation in remote sensing [48], including green spaces in urban environments such as parks and gardens. These vegetated areas can create favorable conditions for *Ae. aegypti* breeding by retaining rainwater or offering shaded sites for rest [49, 50]. The NDWI is calculated using the equation $NDWI = GREEN - NIR / GREEN + NIR$ where GREEN corresponds to the reflectance measure in Band 2 [51, 52]. The NDWI is effective in detecting water bodies in urban environments [53], including stagnant water in basins, pools, and canals, providing insights into potential breeding sites for *Ae. aegypti* [30].

#### Vegetation and buildings

Urban vegetation presence was extracted using the NDVI calculated on the Pléaides image, applying a threshold of 0.20 to keep only the vegetation [54]. The layer related to individual buildings was provided by the French national geographic agency IGN (BD TOPO^®^ 2022), based on cadastre and satellite image photo-interpretation. A masking technique was applied to determine the heights of vegetation and buildings and thus provide information on the vertical structure of urban landscape elements. This involved overlaying the vegetation and building layers with a 2015 LIDAR-derived digital elevation model (1-meter spatial resolution) obtained from CTG. The process filtered out only the elevation information for areas where vegetation or buildings are present.

### Extraction of analysis variables

#### Spatial units of analysis

The following analyses were carried out over a 200 m regular grid cell to ensure sufficient spatial resolution for capturing the diverse characteristics of the urban landscape. QGIS was used to create a regular grid and the R Stats software v. 4.2.1 [55] was used to generate breeding sites variables and urban landscape variables for each cell of the grid. This choice will be discussed in the Discussion section.

#### Breeding site variables

For each grid cell, the absolute number of potential breeding sites (regardless of breeding site types and categories) was calculated by summing positive and negative sites. This was also done by considering separately the different types and categories of breeding sites. Using an analysis grid helps reduce the variations in sampling effort associated with the dataset [56]. A normalized number of potential breeding sites was also calculated by dividing the absolute number of potential breeding sites by the number of prospected locations per cell (Additional file 1). Only modeling performance using the absolute number of potential breeding sites is presented in the Results section due to the similarity of the results.

#### Urban landscape variables

Summary statistics of urban landscape variables were computed within each grid cell: Mean NDVI, Mean NDWI, Mean Vegetation Heights, Mean Buildings Heights, Mean Textural component 1, 2, and 3. The building density (number of buildings per grid cell), and the average building size per cell (total building surface divided by the number of buildings in each cell) were computed using the building layer.

Landscape metrics have been calculated over vegetation and building layers within each grid cell to capture information about urban landscape composition and configuration. This helped quantify the spatial organization of patterns within a defined geographic area. Specific landscape metrics (see Table 2) have been selected based on their potential to relate to the urban landscape and breeding sites of *Aedes* mosquitoes [7, 57]. Thus, by analyzing these several landscape metrics describing the configuration of urban landscape, we could identify links between description of urban landscapes and the spatial repartition of potential breeding sites. For example, more fragmented vegetation could enhance the number of potential breeding sites. In this study, landscape metrics were calculated using the *“landscapemetrics”* R package [58]. Table 3 lists the satellite-derived and geospatial data, their sources, the methods used to create them and the urban landscapes variables derived from them in this study.

**Table 2:** Landscape metrics used in this study [58]

Landscape metrics	Information type	Description	Units	Interpretation
Total class area (CA)	Area and Edge metric	Dominance of one class over the defined area	Hectare	Absolute measure of composition
Largest patch index (LPI)	Area and Edge metric	Percentage of the landscape covered by the corresponding largest patch of each class	Percentage	Measure of dominance
Total class area percentage (CPLAND)	Core Area metric	Percentage of the core area of class to the total landscape	Percentage	Relative measure of composition
Landscape shape index (LSI)	Aggregation metric	Compactness of the class based on the length of the class within the spatial unit	No units	Patch distribution (compact or dispersed)
Patch density (PD)	Aggregation metric	Number of patches of a class within the spatial unit	Number for 100 hectares	Fragmentation
Mean shape (SHAPE_MN)	Shape metric	Ratio between the actual perimeter and the hypothetical minimum perimeter of the patch	No units	Complexity of patch shapes
Mean of Contiguity (CONTIG_MN)	Shape metric	Assigning an adjacency value to each pixel in a patch according to a nine-cell focal filter matrix. Larger and more connections between patch result in larger contiguity index values	No units	Connectivity

**Table 3:** Geospatial data, source information, and variables extracted for urban landscape characterization

Satellite-Derived and Geospatial Data	Information source	Method for layer creations	Remarks	Variables extrated by grid cell	Category of explanatory variable
Pléaides Multispectral	Multispectral	Equations		Mean NDVI	Spectral Indices
				Mean NDWI	
Pléiades Panchromatic	Panchromatic	Algorithm FOTOTEX		Texture PC1	Textural Indices
				Texture PC2	
				Texture PC3	
Vegetation	NDVI threshold	Landscape metrics		Class area	Landscape metrics over vegetation
				Percentage of class	
				Landscape Shape Index	
				Landscape Patch Index	
				Patch Density	
				Mean Shape Index	
		Masking	With MNH from CTG	Mean Height Vegetation	Heights
Buildings	BD TOPO			Mean Height Buildings	
		Landscape metrics		Class area	Landscape metrics over buildings
				Percentage of class	
				Landscape Shape Index	
				Landscape Patch Index	
				Patch Density	
				Mean Shape Index	
		Counting buildings over each grid cell		Number of buildings	Buildings informations
		Total area of buildings divided by the number of buildings for each grid cell		Mean size of buildings	

### Multiple factor analysis

Multiple Factor Analysis (MFA), a multivariate statistical technique, was performed to explore and analyze relationships among multiple quantitative and/or qualitative datasets [59]. The analysis consists of grouping variables, depending on study objectives, and in balancing the contribution of the groups in the analysis – i.e. in compensating the contribution differences due to variable contribution themselves and/or to the number of variables per group. This approach aims to favor the identification of the links between variables, within groups (like a classical principal component analysis (PCA) – if variables are quantitative, as it is the case here) but also between the different groups. As for PCA, the factorial axes represent the main orthogonal directions along which the data vary the most and the variable contributions indicate to which extent each variable contributes to the factorial axes (the sum of the variable contributions for a given axis is equal to 1), while the squared cosines (cos²) measure to what extent the variables are well represented on each axis (the sum of the cos^2^ of a given variable over all factorial axes is equal to 1). This analysis aims to identify variables characterizing urban landscapes that are the most discriminant regarding variables characterizing *Ae. aegypti* breeding sites, and to select some of them for modeling purposes. This approach was applied to two groups of variables: one grouping the 24 breeding site variables (i.e., number of potential breeding sites, number of potential breeding sites of each type and regrouped category; Table 1) and another one grouping the 24 urban landscape variables (i.e., spectral indices, textural indices, landscape metrics over vegetation and landscape metrics over buildings, heights, and buildings information) (Table 3). In that way, we can identify if some types of breeding sites were associated with specific urban landscape variables. The selection of urban landscape variables for modeling relies on identifying variables with contribution values above the average contribution and squared cosine (cos²) values above 0.1, considering the first three factorial axes. Then a selection of one variable per group of explanatory variables was performed to do the prediction. In this study, MFA was conducted using R “*FactorMineR*” package [60].

### Modeling and analysis

Non-linear models were used to establish the relationships between different types of potential breeding sites (dependent variable) and urban landscape variables (independent variables). We applied Random Forest (RF), which combine multiple decision trees for regression [61]. RF can model complex relationships between explanatory variables and the target variable with great robustness, allowing to deal with non-linearly separable data (more precisely, it is a “piecewise linear model”). RF is a non-parametric model that imposes no limitations and makes no assumption about the distribution of the data, allowing greater flexibility [62]. It showed its efficiency for regression and prediction applied to spatio-temporal distribution or abundance of *Ae. aegypti* (see *Introduction* and [37, 38]). However, its application to potential breeding sites has not been explored yet. Different RF models were built, considering different explanatory variables grouping: variable categories indicated in Table 3 (texture indices, spectral indices, heights, landscape metrics related to vegetation, landscape metrics related to buildings, building information); variables selected according to MFA (referred to as MFA variables hereafter); and the complete set of variables. A range of response variables were tested, which included all potential breeding sites, as well as all types and categories of potential breeding sites (cf. Table 1). Models were applied to grid cells that include a minimum of five home visits, resulting in a total of 417 grid cells (51% of the grid cells with at least one record) analyzed. This threshold of five home visits was chosen to ensure that the analyzed grid cells had a sufficient sample size for reliable and robust statistical analysis. In this study, RF models were computed using R “*randomForest*” package [63].

To assess the RF models’ performance, a cross-validation procedure was applied, by performing a random selection of 70% of the 417 grid cells to build the training set and keeping 30% of the cells to test the model. Such a procedure was repeated 100 times, with replacement of the cells for the random selection (bootstrapping). For relatively rare breeding site types that are only present in a few cells of the study area, such a procedure can lead to building models with too few cells. Consequently, we made sure that each individual model was based on enough cells with a presence of the specific type This was done by removing cross-validation iterations that used, for training, less than 70% of the cells where the considered breeding site type or category was observed.

**Fig. 2:**
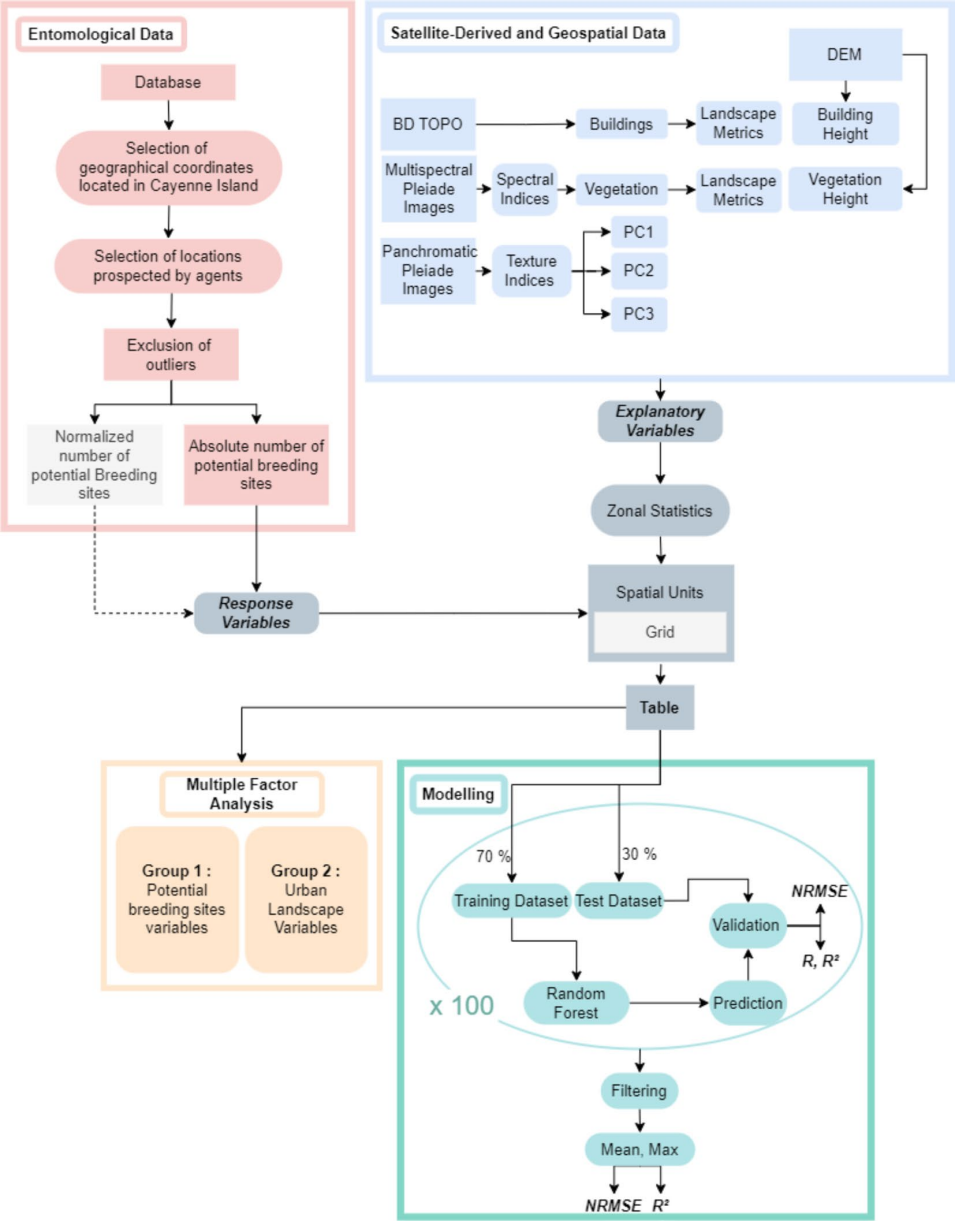
Methodological framework of this study

The Normalized Root Mean Square Error (NRMSE) was computed to evaluate the prediction quality while reducing the impact of extreme values and considering the overall distribution of values in the data. As the response variables did not follow a normal distribution, the NRMSE based on a min-max range was used to incorporate a measure of spread of the data. The NRMSE provides a relative measure of the model’s effectiveness, where a low NRMSE value indicates a more accurate fit of the model. The coefficient of determination (R²), was used to assess the performance of regression models over observations and results of predictions made on each validation sample [64]. Figure 2 summarizes the methodological framework of this study.

## Results

### Descriptive analysis of entomological data

#### Sampling effort

**Fig. 3:**
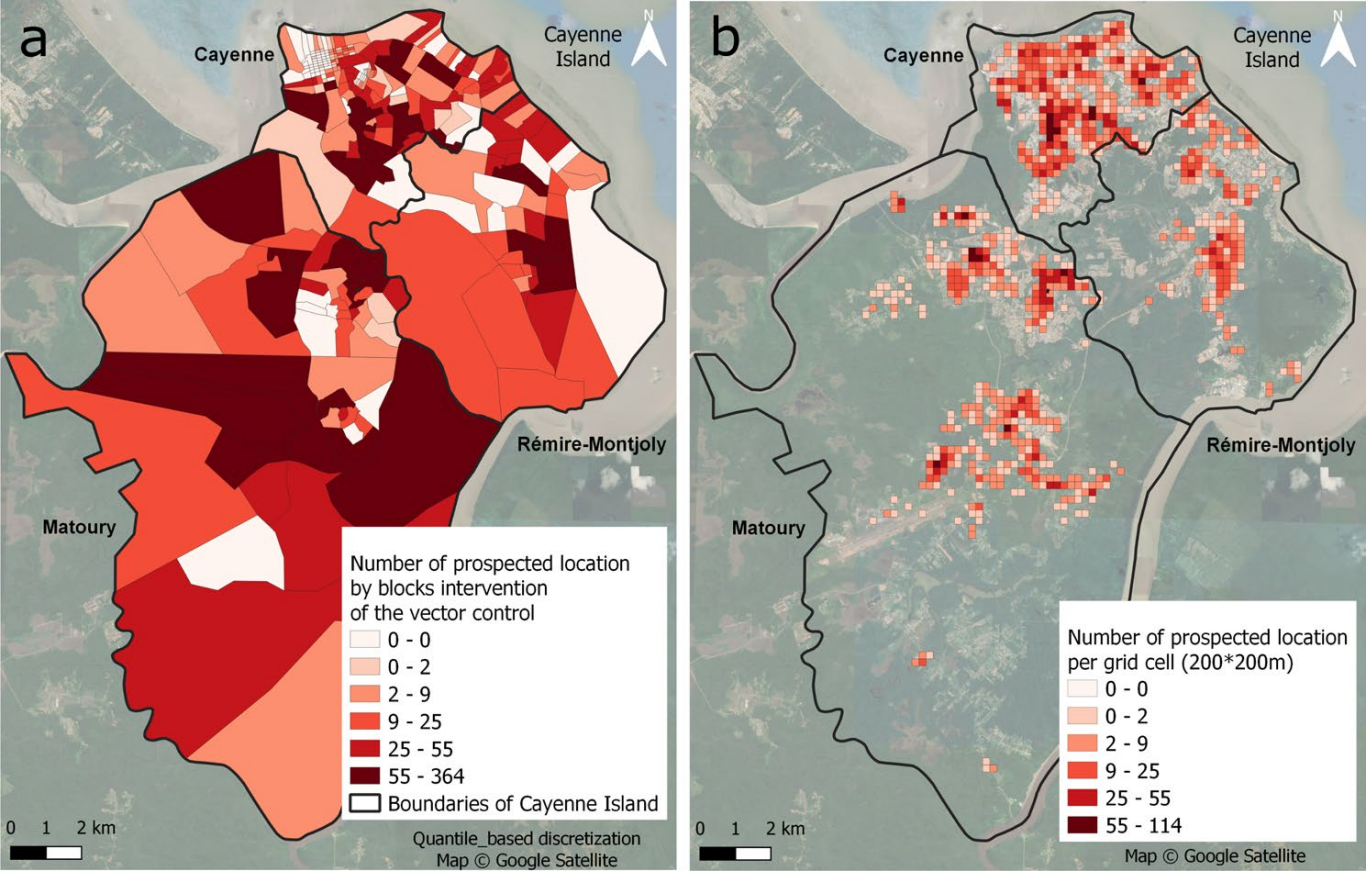
Entomological data sampling effort over (**a**) blocks, and (**b**) grid cells for 2022 on Cayenne Island

The number of locations visited by vector-control agents, when aggregated by survey block, ranged from 0 to 364, with an average of 30 homes (Fig. 3a). When aggregated by grid cells (Fig. 3b) excluding cells with no visited houses, sampling effort ranges from 1 to 114 houses, with an average of 10 homes.

#### Potential breeding sites

**Fig. 4:**
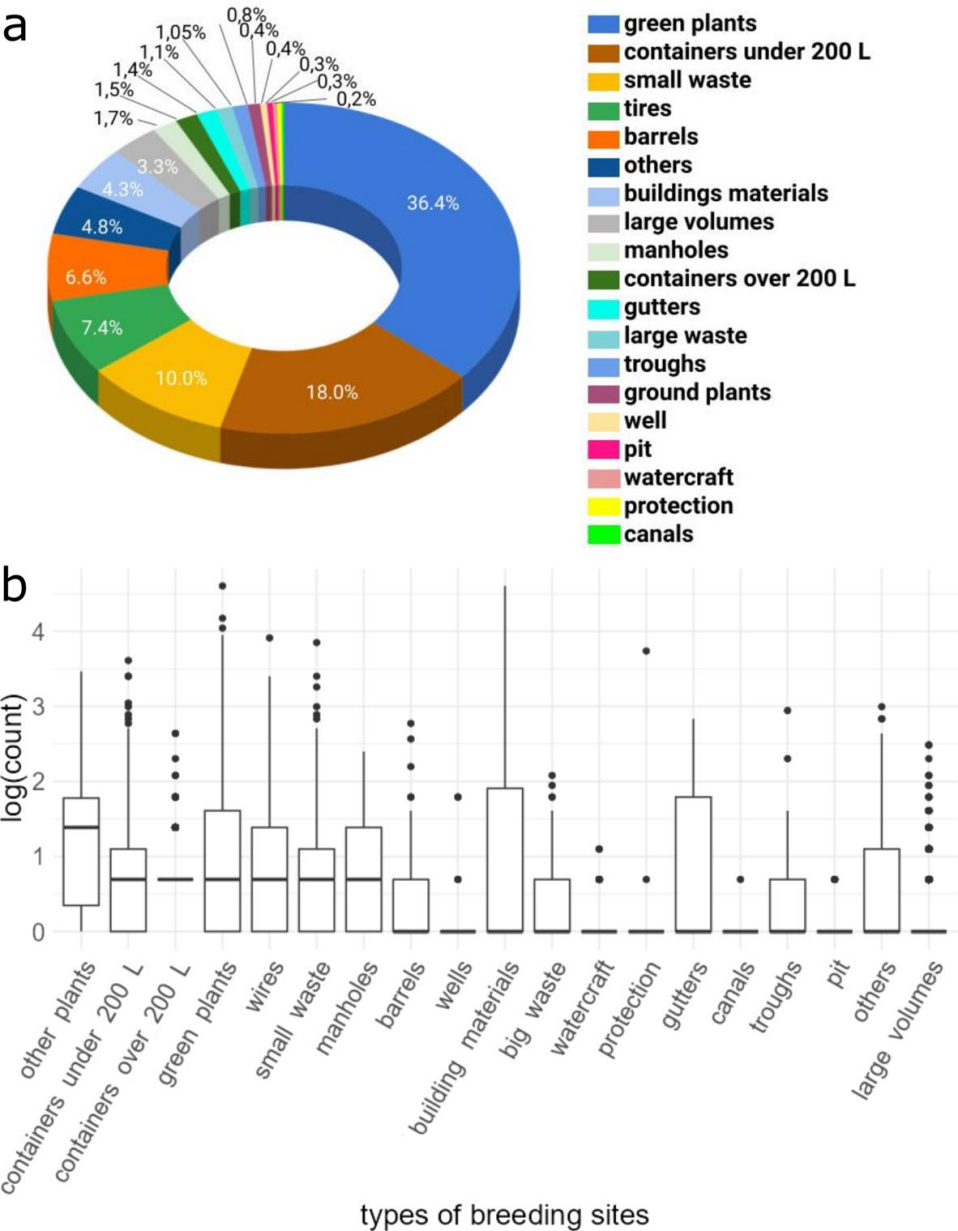
(**a**) Proportion of breeding sites per type in Cayenne Island; (**b**) Logarithmic count of potential breeding sites, categorized by types and ordered by median value

**Fig. 5:**
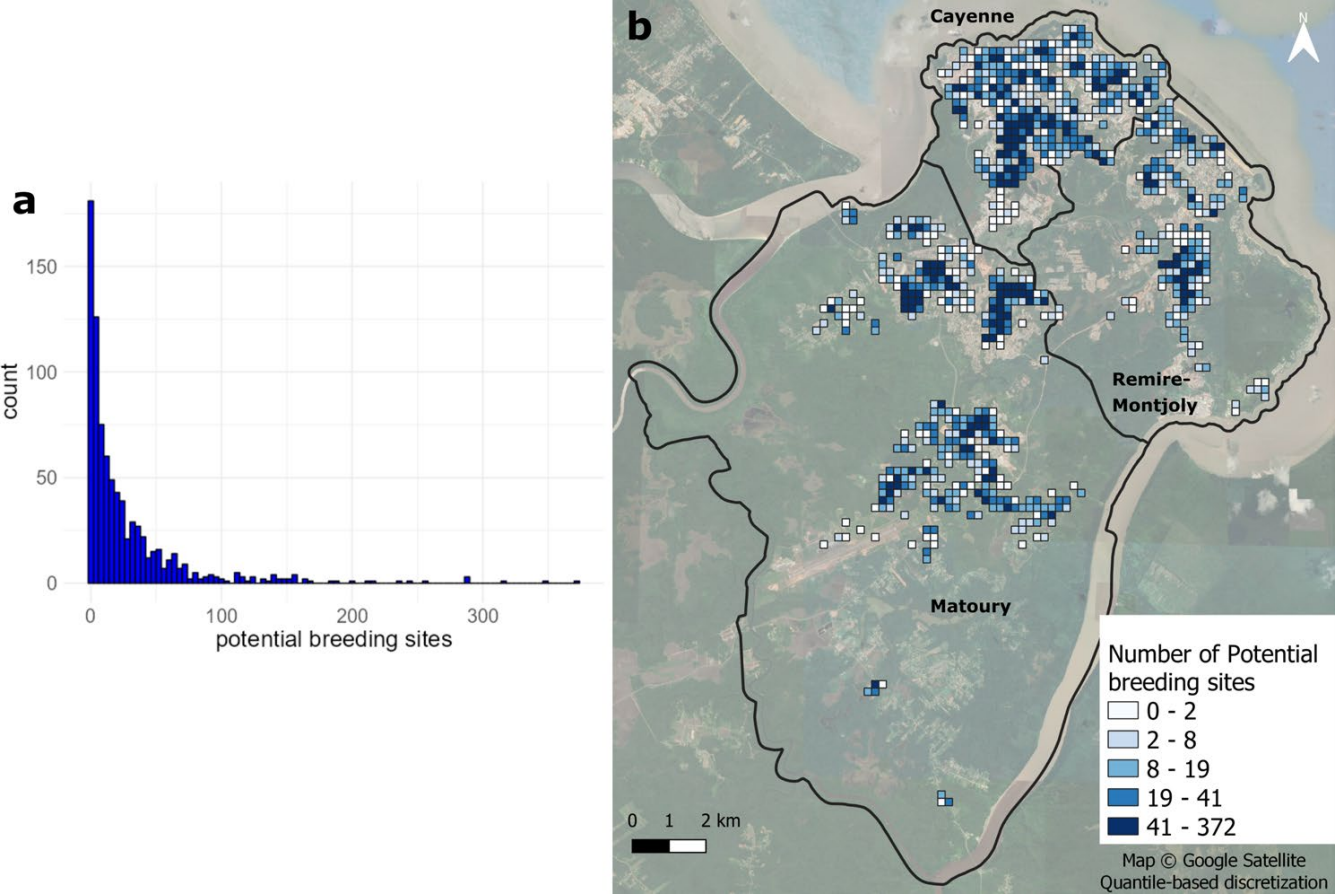
Number of potential breeding sites per grid cell as (**a**) a histogram and (**b**) geographically

Analyses revealed a strongly positively skewed distribution of potential breeding site numbers, with a mean and a median of 2.68 and 1 sites per house, respectively. On Cayenne Island, 5 of the 19 breeding site types, account for 78.4% of all breeding sites: green plants (36.4%), containers under 200 L (18.0%), small waste (10.0%), tires (7.4%), and barrels (6.6%) (Fig. 4a). Both the median and the third quartile of the logarithmic number of potential breeding sites are very low, ranging from 0 to 1.4 and 0 to 1.95, respectively, regardless of the type. Building materials or green plants present the highest maximum values of potential breeding sites (Fig. 4b). The number of potential breeding sites per grid cell also display a strongly positively skewed distribution (Fig. 5a), with a mean absolute value of 28, a maximum of 372, and a median of 13. The spatial representation of the number of potential breeding sites per grid cell (Fig. 5b) shows higher values over residential areas in Cayenne, Matoury, and Remire Montjoly in contrast with lower values in peri-urban areas of these towns.

### Extraction of analysis variables

All extracted variables are represented geographically in Additional files 2, 3, 4 and their histogram is provided in Additional file 5.

#### Textural indices

**Fig. 6:**
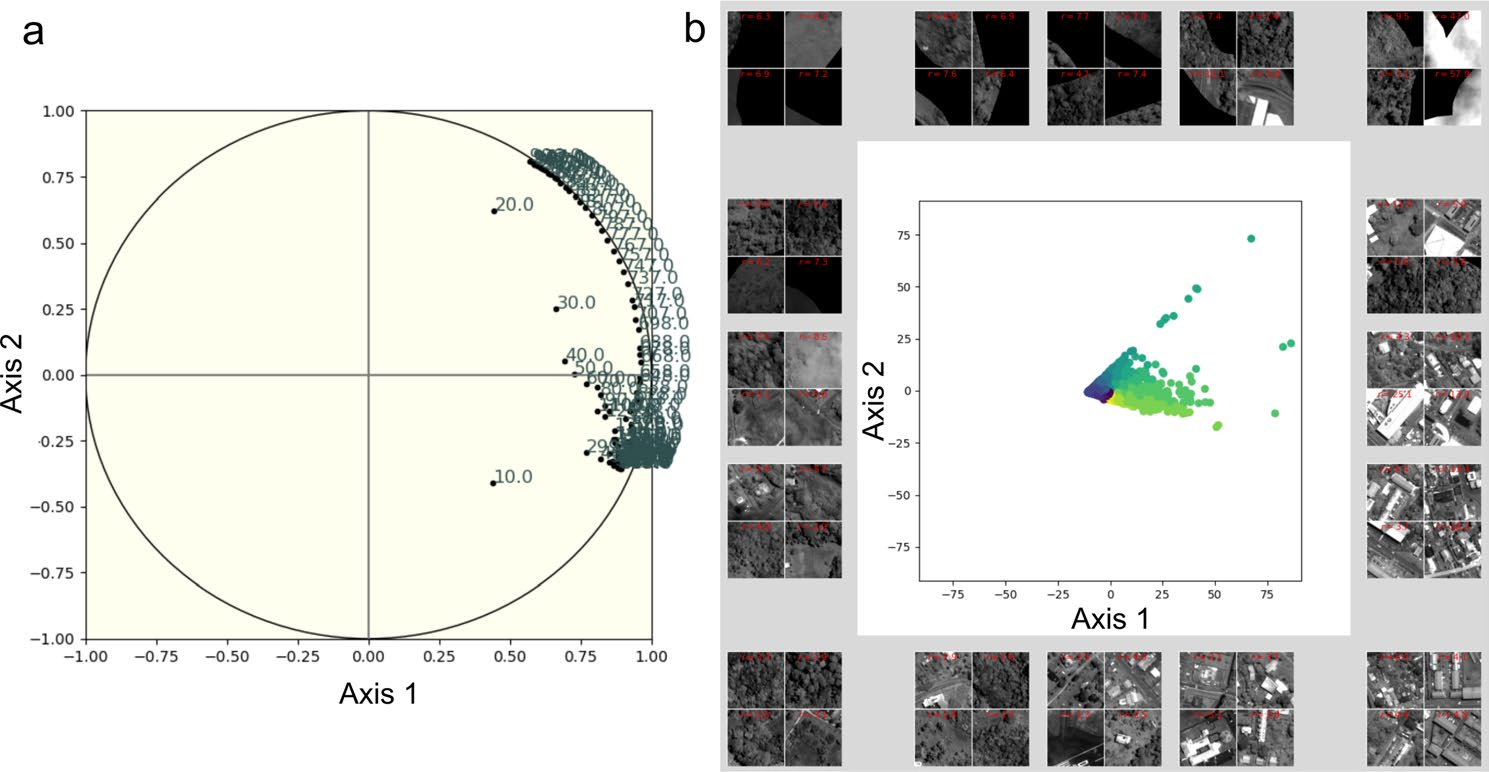
(**a**) Representation of the spatial frequencies (expressed in cycles.km^− 1^) on the 1st factorial plane defined by the first two factorial axes; (**b**) Analysis window projections on the first factorial plane defined by the first two factorial axes. Pléiades © CNES 2022 Distribution Airbus DS

**Fig. 7:**
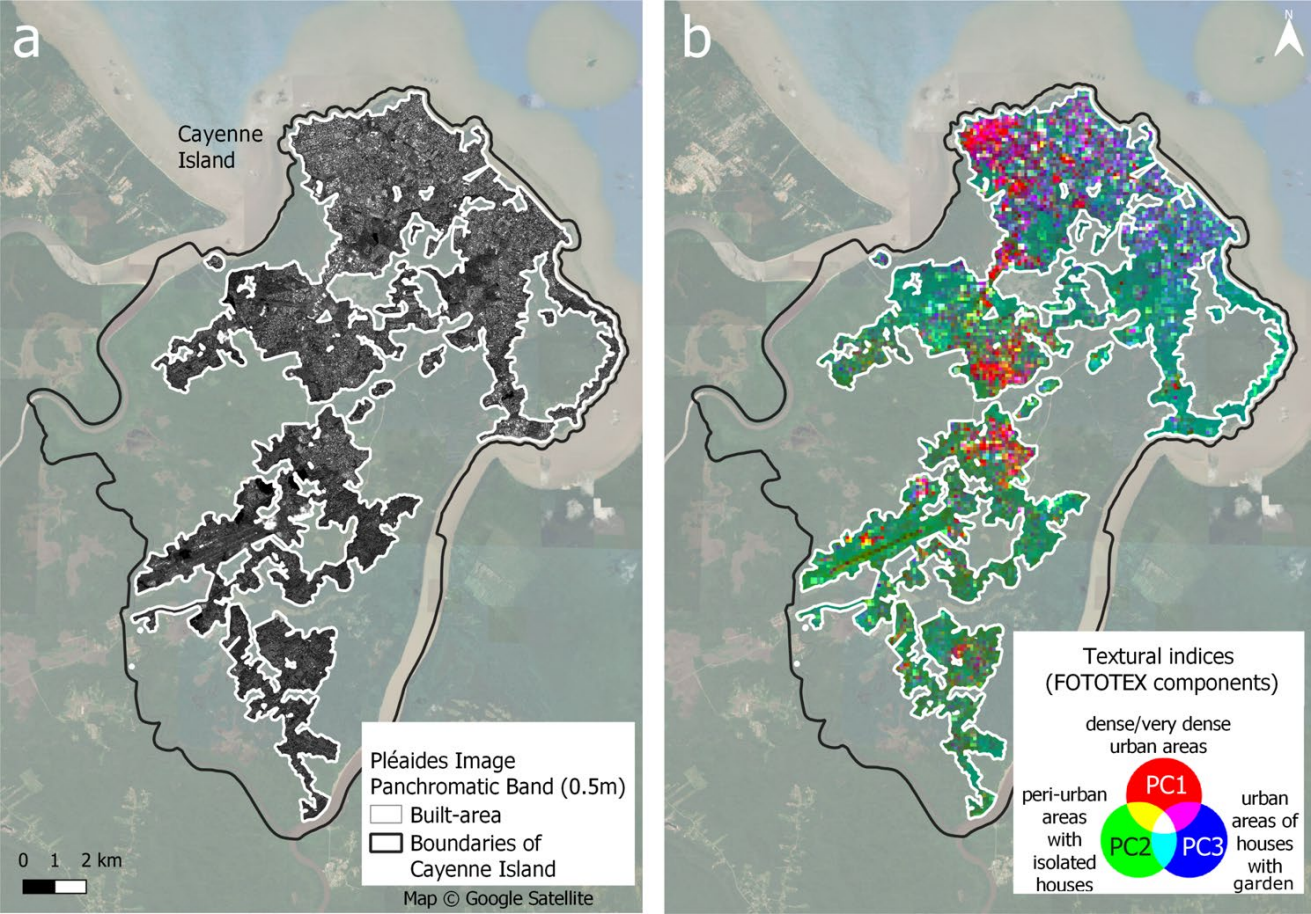
(**a**) Panchromatic band of the Pléiades image where built-up areas have been delineated and FOTOTEX has been applied. (**b**) Textural indices produced by FOTOTEX: RGB composite images with the first three Principal Components (PC) - Component 1 in the red band (69% of variance), C2 in the green band (18%), and C3 in the blue band (0.03%). Pléiades © CNES 2022 Distribution Airbus DS

FOTOTEX analyses resulted in three principal component analysis (PCA) axes, accounting for 87.03% of the total variance of the image (i.e., 69%, 18% and 0.03% respectively). Frequencies are concentrated along the value 1 of the first axis (Fig. 6a). The second axis displays frequencies ranging from intermediate to high values, even reaching very high frequencies (996 cycles.km-1). The relationship between specific urban landscapes and their position along the two PCA axes is illustrated in Fig. 6b. By analyzing the windows with the most extreme values along the bisector in each angular sector (corresponding to a section of a given angle of the factorial plan), we observe that windows with urban patterns are present close to values of 1 on the Axis 1 and along the Axis 2. FOTOTEX RGB composite (Fig. 7a, b) allows for a rapid assessment and overview of the spatial organization of urban landscapes based on Pléaides imagery. Different types of urban areas are well-discriminated, especially with dense or very dense urban areas in pink/red, areas gathering houses with gardens in purple/blue, and peri-urban areas with isolated houses dominated by the presence of vegetation in green (Fig. 7b).

### Landscape metrics

#### Vegetation

**Fig. 8:**
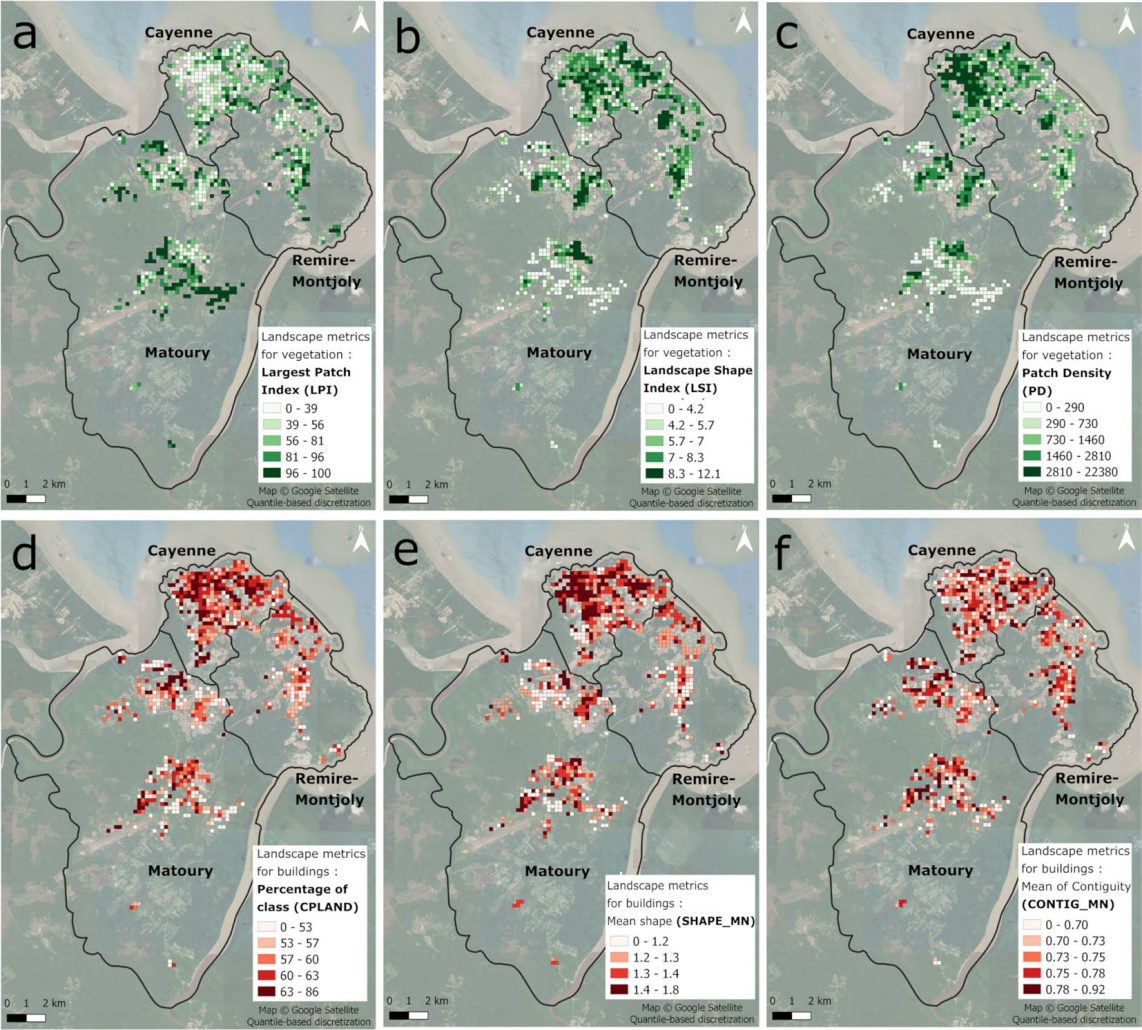
Landscape metrics applied to vegetation and buildings (**a**) Largest Patch Index (LPI); (**b**) Landscape Shape Index (LSI); (**c**) Patch Density (PD); (**d**) Percentage of class (CPLAND); (**e**) Mean Shape (SHAPE_MN); and (**f**) Mean of Contiguity (CONTIG_MN)

When applied to vegetation, the total class area, percentage of the class area, and the largest patch index metrics all collectively show an increasing surface of vegetation from the center of Cayenne towards the neighboring towns of Remire-Montjoly and Matoury, closer to forested areas (Fig. 8a). Landscape shape index suggests that Cayenne’s vegetation has a higher connectivity between patches than the south of Matoury (Fig. 8b). Patch density indicates higher fragmentation, especially in Cayenne’s center, north of Remire-Monjoly, and in four clusters in Matoury (Fig. 8c). Mean shape shows that Remire Montjoly and Matoury have on average higher values per grid, indicating a higher diversity of patch shapes than in Cayenne.

#### Buildings

Class area, and percentage of class show a greater presence of the building class in Cayenne and in the residential areas of Matoury than in Remire Montjoly and in the peri-urban areas bordering the forests in Matoury (Fig. 8d). Largest patch index indicates a pronounced presence of largest patches of buildings in Cayenne, north of Matoury and in peri-urban areas in South Matoury. Landscape shape index shows more compactness of buildings, in Cayenne and the residential areas of Matoury. Then patch density shows a greater fragmentation in Matoury and Remire Montjoly than in Cayenne. Mean shape indicates higher diversity of patch shapes, especially in Cayenne and residential areas of Matoury (Fig. 8e).

Finally, mean of contiguity shows strong heterogeneity of values across the landscape, indicating a challenge in identifying clear connectivity patterns among grid cells for vegetation and buildings (Fig. 8f).

### Multiple factor analysis

**Fig. 9:**
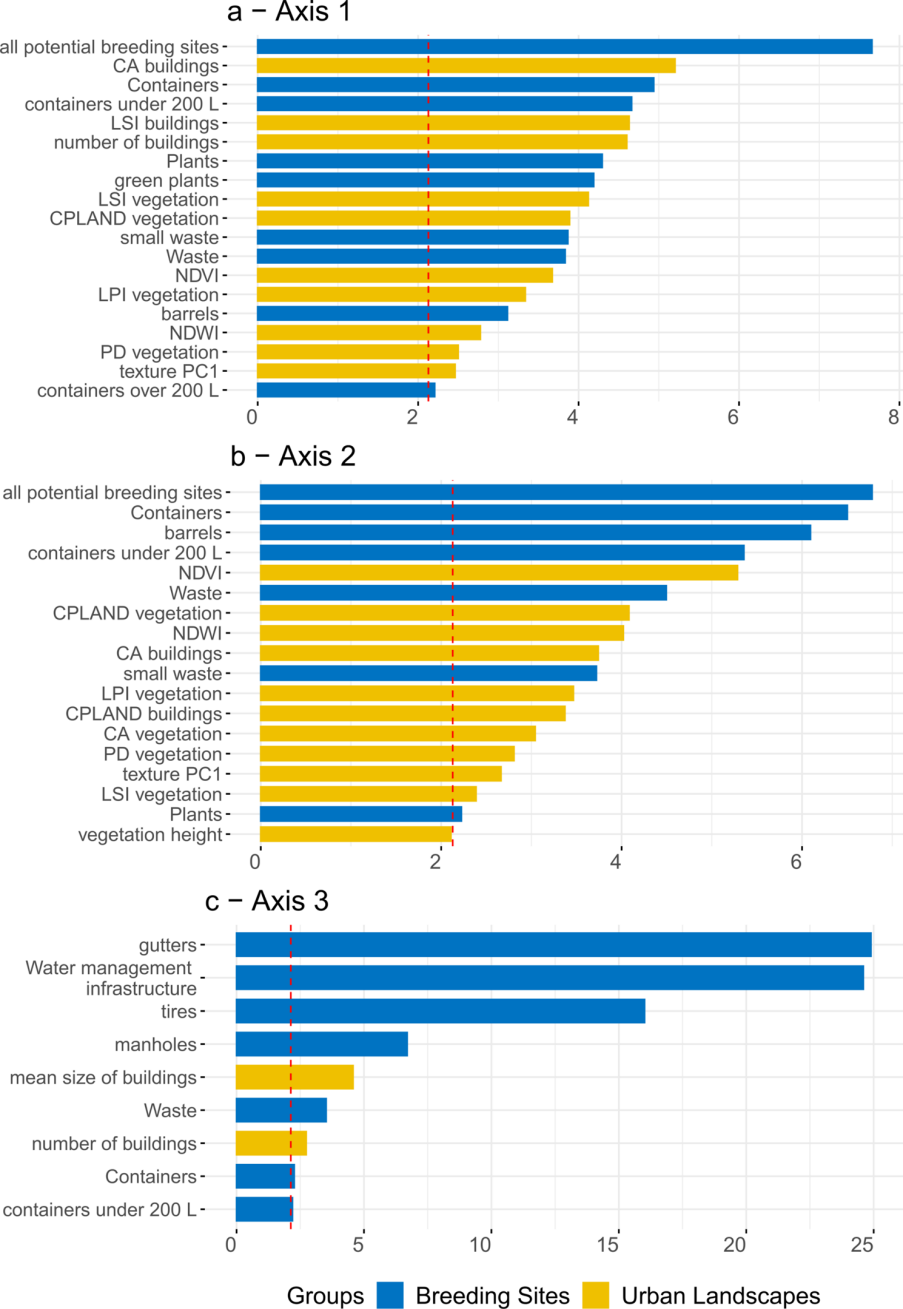
Contributions of quantitative variables above the mean contribution of each axis (red dashed line) to (**a**) Axis 1 (**b**) Axis 2 and (**c**) Axis 3. CA: Total Class Area, CPLAND: Total class area percentage, LPI: Largest patch index, LSI: Landscape shape index, PD: Patch density, SHAPE: Mean shape, CONTIG: Mean of Contiguity

MFA three principal axes account for 40.8% of the total variance (21.9%, 12.4%, and 6.5% respectively for Axes 1, 2, and 3). Variables considered as significantly contributory to the first three axes (i.e. with contributions above the mean contribution of all variables for each axis) are presented in Fig. 9.

#### Axis 1

Landscape variables with the most influence on variance are total building class area and landscape shape index (vegetation and buildings), along with the number of buildings and the percentage of vegetation (Fig. 9a). NDVI, largest patch index, NDWI and the first component of texture indices (texture PC1) also significantly contribute to the first axis. In contrast, for breeding site variables, the potential breeding sites is the most contributory variable, followed by potential breeding sites of the categories containers and plants. These are followed by three specific types of breeding sites: containers under 200 L, green plants and small waste.

#### Axis 2

The potential number of breeding sites is also the most contributory variable, followed directly by NDVI (Fig. 9b). The next four contributory variables are breeding site-related variables (i.e. all potential breeding sites, containers, barrels, containers under 200 L). Variables associated to vegetation, buildings and the breeding sites “waste”, “small waste” and “plants” are less contributory. The first component of texture is in the 9th position for urban landscape variables. Notably, vegetation height is part of the most contributory variables on Axis 2.

#### Axis 3

Mean building size and number of buildingsare the most contributory urban variables (Fig. 9c).

**Fig. 10:**
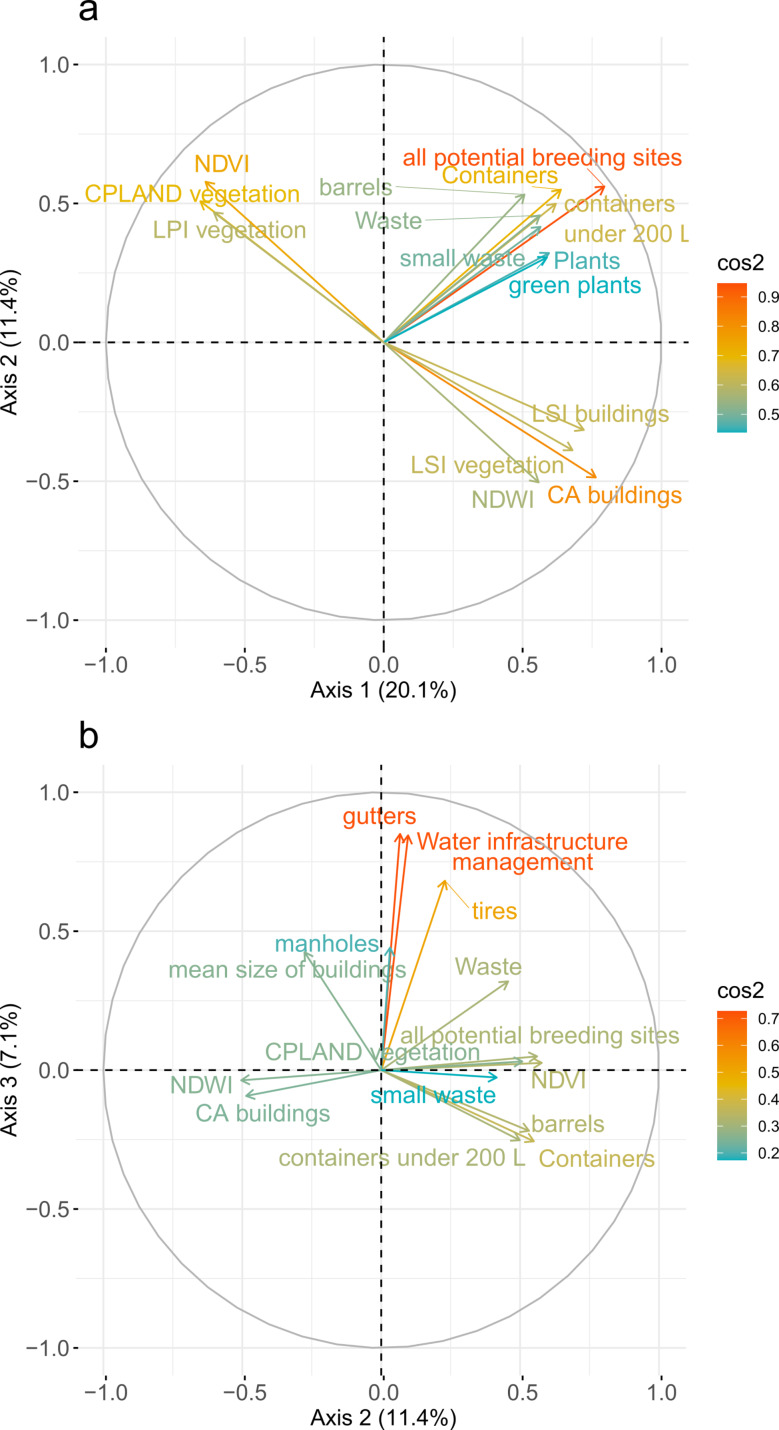
Variables, with quality of the representation (cos^2^) represented by the arrow color, and correlation circle, for factorial plans defined by (**a**) Axes 1 and 2 and (**b**) Axes 2 and 3

The representation of the variables on the factorial plans allows identifying positive correlations when variables are clustered within the same quadrant and negative correlations when variables are positioned in opposite quadrants relative to the axes’ origins (Fig. 10a). Consequently, three groups can be distinguished: one made of urban landscape variables positively correlated with Axis 1, another made of urban landscape variables positively correlated with Axis 2, and a third group containing breeding site variables, perpendicular to the urban variable groups. On the correlation circle with Axes 2 and 3, potential breeding sites appear to be correlated with the percentage of vegetation, but with low representational quality (Fig. 10a).

Consequently, for RF models, the selected variables were building class area, number of buildings, vegetation landscape patch index, NDVI, NDWI, the first component of texture, and the height of vegetation.

### Modeling results

**Fig. 11:**
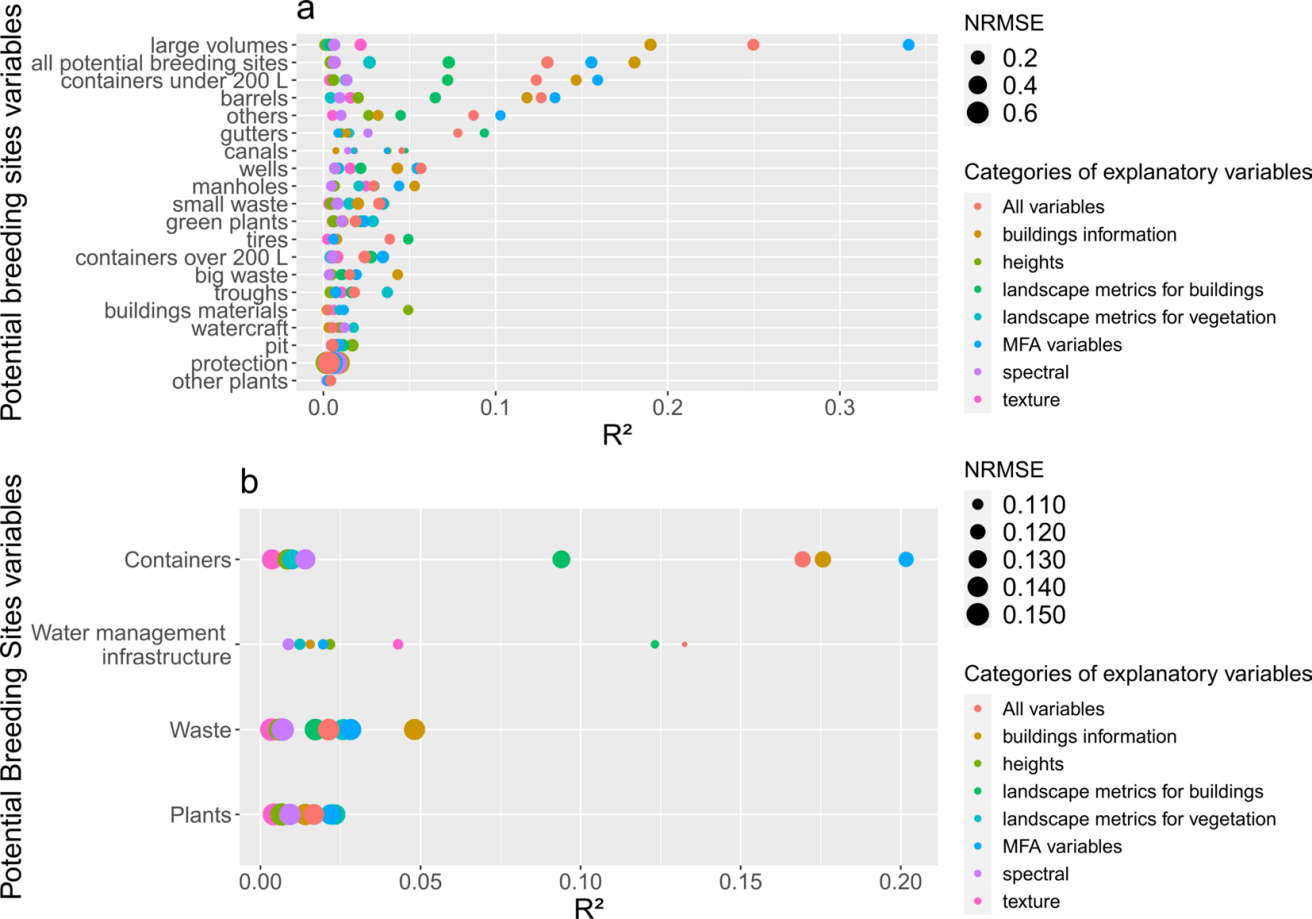
RF models mean R² and NRMSE values when considering different response variables and different groups of explanatory variables for (**a**) types of potential breeding sites, and (**b**) categories of potential breeding sites

Random Forest (RF) models provided mean R² values ranging from 0 to 0.20 for the categories of breeding sites (Fig. 11a) and from 0 to 0.33 for the types of breeding sites (Fig. 11b). The average NRMSE between observations and predictions is 0.14 (range: 0.05–0.65). Detailed analysis of the RF models for potential breeding sites shows that the “large volume” breeding sites type has the highest average prediction result with “MFA variables’’, with an R² of 0.33 and an NRMSE of 0.13 (Fig. 11a). When using the variables selected from the MFA, the R² for “all potential breeding sites” is 0.15, while it is 0.18 when using only “buildings information”, with the NRMSE both at 0.14. “Containers of less than 200 liters” have a R² of 0.16 and an NRMSE of 0.11 with “MFA variables” and with only “buildings information” a R² of 0.14 and NRMSE of 0.12. Next, the category of “Containers” grouping several types of breeding sites provide an R² of 0.20 and an NRMSE of 0.11 (Fig. 11b). The categories of explanatory variables with the highest R² are “building information” and “MFA variables”, except for category “Water Management Infrastructure” and for types “gutters”, “canals”, “tires”, “troughs”, “buildings materials”, “watercraft”, “pit”, and “other plants”. Texture, heights and landscape metrics, when taken individually, provide low predictive powers (R² below 0.10). Some types of breeding sites like “gutters”, “canals”, and “tires” have higher R² values with “landscape metrics for vegetation” variables but these values remain low (between 0.05 and 0.10). Many types of breeding sites have very low R² values (below 0.02) and rather higher NRMSE values: “others plants”, “protection”, “pit”, “watercraft”, and “buildings materials”. Overall, there is considerable variability in model performance across breeding sites types and categories, with both low R² values and NRMSE values. When analyzing the maximum performance values of the RF models (Additional file 6), for “large volumes” the R² is 0.94 with “buildings information”, closely followed by MFA variables, although NRMSE are higher (around 3). For “all potential breeding sites” or “barrels”, the maximum R² is 0.35 with “buildings information” and NRMSE values are under 1, regardless of the predictor variables considered.

**Fig. 12:**
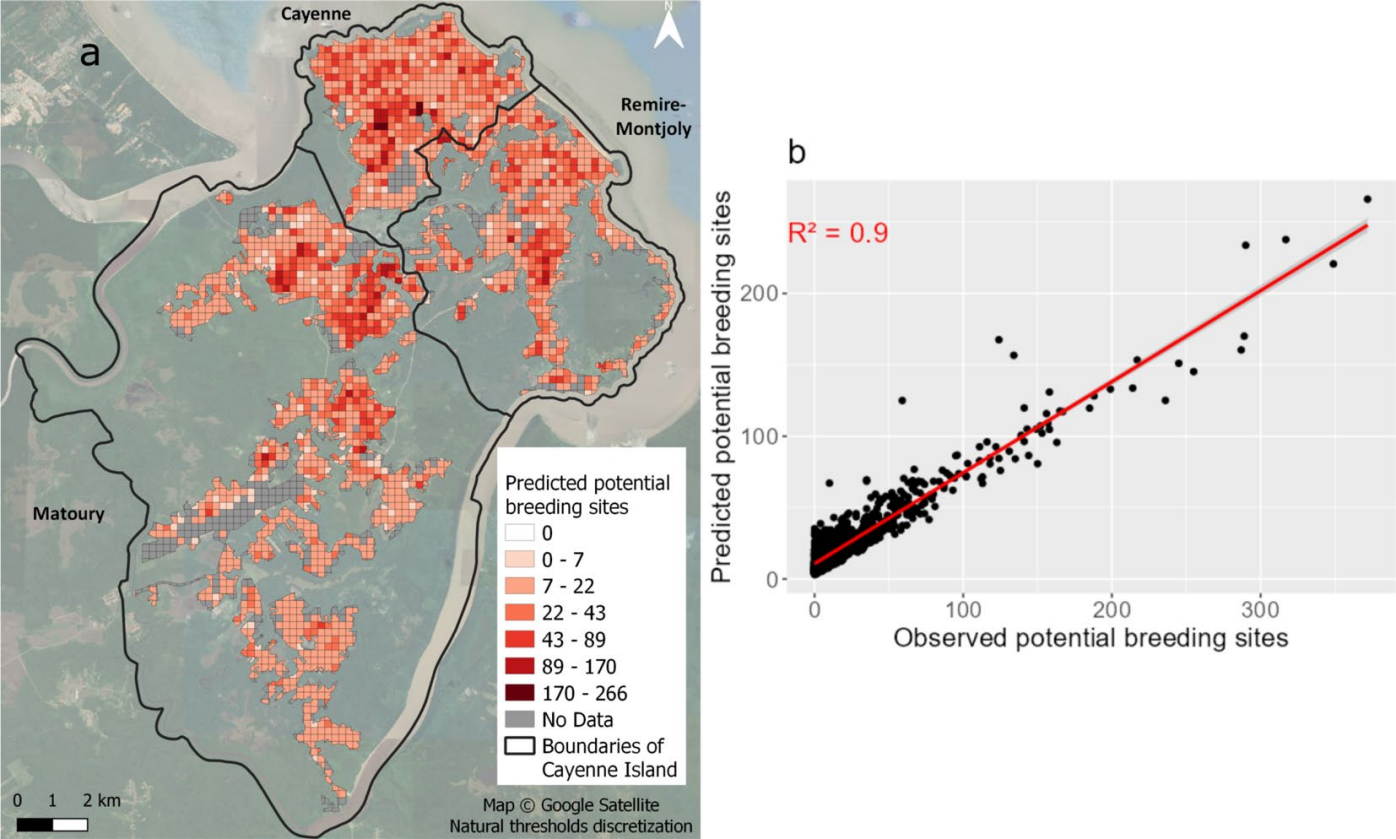
(**a**) Predicted values of potential breeding sites per grid cell for Cayenne Island; (**b**) Observed vs. predicted values of potential breeding sites from the RF model using MFA variables for the full dataset

When a model was trained on the complete dataset with the set of variables selected based on MFA, the number of potential breeding sites can be predicted for the entire urbanized area of the Cayenne Island (Fig. 12a). In this case, the R² between the observed values used for the model and the predicted values is 0.90 (Fig. 12d). Residential areas with high densities of buildings are the areas where predicted potential breeding sites are higher (over 43 per grid cell) while commercial areas concentrate less predicted potential breeding sites (under 22 per grid cell). Peri-urban areas with isolated houses show values between 7 et 22, with some areas exhibiting numbers of potential breeding sites above 43. The model did not predict any cells with zero potential breeding sites.

## Discussion

This study aimed to better understand how the distribution of *Aedes aegypti* mosquitoes’ potential breeding sites relates to urban landscapes, with the goal to assess to what extent urban environmental variables can spatially predict the number of breeding sites. Targeting specifically potential breeding sites, our study tries to assess the number of available breeding sites in a given territory. In fact, as the presence of larvae depends on many factors and is not always easy to establish in the field, predicting positive breeding sites only can result in underestimating the actual capacity of charge of the environment, i.e. the capacity of the territory to “produce” mosquitoes. We consider that to improve vector control strategies, it is interesting to focus on potential breeding sites rather than positive ones to better estimate breeding sites availability and target priority areas during inter-epidemic periods, or optimize efforts and resources during epidemic periods. Moreover, mechanistic models like the one developed by Tran et al. [65] require the capacity of charge of the environment as an input, corresponding, in practice, to the number of potential breeding sites per surface unit (e.g., per ha).

The French Guiana entomological data proved to be heterogeneous, with a high variety of breeding site types and categories over the study area (Figs. 4 and 5, Additional file 8). MFA performed using quantitative variables did not reveal strong linear relationships between urban and potential breeding site variables. However, the factorial plane defined by the axes 2 and 3 of the MFA shows a more contrasted distribution of variables than the one defined by the axes 1 and 2 (Fig. 10), which led us to use the MFA to select the most contributory variables to build the prediction model. Moreover, to take advantage of the non-linear relationships between variables related to breeding sites and environmental variables, a RF algorithm was implemented to see if potential breeding sites could be predicted. We showed that the selected variables from MFA and the set of variables related to buildings resulted in the best performances of the RF models for predicting the number of all potential breeding sites (Fig. 11a). This can be explained by the fact that these models integrate the number of buildings and the mean building size, variables which have a strong link with the number of potential breeding sites [66]. In the present study, the other environmental variables did not appear significantly linked to potential breeding sites (as a whole or considered by type or category). The low performance values ​​of RF models over several types of potential breeding sites could be explained by the strong variability in the data. Even when applying a 70/30 split with bootstrapping for cross-validation, and by taking into account a minimum of non-null cells for training, the quantity of data used by the models is very variable depending on the types of breeding sites. Among the 417 cells used in the modeling process, only 20 have canals, 23 with protection devices (like tarpaulin), 39 with watercraft, 50 with pits, and 21 with “other plants” (not in pots). Even with a greater availability in the study area, some types of breeding sites still have model performances that remain very low (small waste, green plants, tires) thus demonstrating the difficulty of identifying predictive variables relevant for all types of breeding sites. We compared the modeling performance using absolute versus normalized number of potential breeding sites (absolute number of potential breeding sites divided by the number of houses visited in each cell) to take into account the sampling effort. We found that no matter the type of breeding sites considered, both R² and NRMSE consistently yielded lower values for normalized (mean range from 0 to 0.17) (Additional file 7) than for absolute number of potential breeding sites.

Our study suggests that although satellite image texture analysis can discriminate between neighborhood types [41], among the texture variables, the first FOTOTEX principal component was the only one significantly contributory, according to the MFA. The first component of FOTOTEX concentrates the majority of the information related to urban texture, accounting for 69% of the variance. It corresponds to a high building densities (red band in Fig. 7), and consequently can be considered as equivalent to the number or buildings derived from BD TOPO database. As such, a variable derived from remote sensing can be produced in any city, making the prediction method presented in this paper reproducible, whereas topographic databases such as BD TOPO are not available in every country. Even though the first component of texture is used in RF models as one of the MFA selection variables and shows one of the best average prediction performances on test sets, performance of texture variables, when considered as one group of explanatory variables, was found to be insufficient.

In our study, unlike in Espinosa et al. [67] and Arduino et al. [33], we focused on the direct relationship between potential breeding sites and landscape descriptors through the use of a regular grid, avoiding the need for a previous segmentation of the urban space based on its characteristics, which is a complex task. We consider that a more detailed and fine-resolution description of urban areas better captures the complexity and heterogeneity of the landscape hence allowing a better estimation of potential breeding site distribution. Albrieu-Llinàs et al. [34] seem to be the only paper that tried to integrate the urban landscape diversity in relation to positive breeding sites, using it to segment the urban areas. In our study, the landscape patch index (LPI) used on vegetation data and the percentage of building class per grid cell, were amongst the most contributory variables, being well represented in the MFA to use in modeling potential breeding site distribution (Fig. 9a). This shows that in urban areas, not only buildings but also urban vegetation contributes to explain the number and distribution of potential breeding sites. This can be explained by the fact that vegetation can support the creation of potential breeding sites, like tree hollows or plants creating water retention [68, 69], and more generally by the fact that vegetation is a key element of the urban landscape characterization. Landscape metrics associated with fragmentation (PD), complexity (SHAPE) and contingency (CONTIG) did not appear to be linked to potential breeding sites in the MFA analysis and also as explanatory predictors in the RF models. Landscape metrics related to composition (CA, CPLAND), dominance (LPI) and compactness (LSI) show more significant relationships with potential breeding site distribution. This study brings the first responses regarding the influence of composition of urban landscapes on the distribution of *Aedes aegypti* breeding sites.

While studies found that *Aedes* mosquitoes can be found on all floors of buildings [70, 71], mosquito density tends to decrease with increasing building levels [72]. We found no study that examined the relationship between the number of potential breeding sites and building or vegetation height. In our study, while vegetation height was contributory and represented in MFA analysis, the height of buildings did not prove significant in explaining or predicting potential breeding sites. This may be attributed to either the absence of a meaningful relationship between them, or too small variations in building heights in the area to have an impact on potential breeding site distribution (value of buildings between 0 and 18.4 m) or to the mismatch between the 2015 digital terrain model data and the 2022 breeding site data.

Climatic variables such as temperature, precipitation and relative humidity were not included in this study for three reasons. First, there is no compelling evidence in the literature of a clear link between those variables and the distribution of potential breeding sites, when compared to variables that characterize urban landscapes [73, 74]. Second, small spatial heterogeneity in temperature and rainfall across the study area would not impact population practices (e.g., water storage, plant watering) and the subsequent potential breeding site presence and abundance. Finally, the spatial resolution of existing climatic data would have been much lower than the resolution of the urban variables that were used in our analyses.

The present study shows that relationships between urban landscapes and potential breeding sites are difficult to identify and characterize, in particular due to the difficulty of extracting variables that effectively capture the heterogeneity of the urban landscapes. The determination of the suitable scale of analysis and of the spatial units for urban landscape variable calculation is another reason why this question is difficult [75]. For potential breeding site data, the 200 m grid appeared to be effective at reducing the influence of outliers and sampling effects. It also helps reduce spatial autocorrelation that occurs when buffers are used around each sampled house. Such a grid is relevant as it permits to work with spatial units (cells) presenting uniform size and form, at a spatial resolution that both permits to work at a local scale, while identifying repetitions of urban patterns. The blocks (i.e. the division of the territory used by vector control agents) (Fig. 3) were considered as spatial units of analysis, but appeared less relevant than grid cells due to strong heterogeneity in block size and shape. Despite the importance of the spatial nature of response and predictor variables (geospatial data), models used in the study did not explicitly incorporate the spatial locations of the grid cells or any other explicit spatial structures (which can be considered through spatial random effect). The explicit integration of variables on spatial structure or potential neighborhood effects could ameliorate the results [76, 77].

Finally, this entomological dataset made of data routinely acquired by vector control services was not designed specifically for such a study. Using this dataset may have introduced biases as they were collected for immediate and efficient operational needs, leading to oversampling or undersampling over specific geographic areas and/or in specific environmental contexts. Despite the relatively low predictive power (R²) of the RF model, the model demonstrated a high goodness of fit (R²=0.9) when using the entire dataset for Cayenne Island. Such a high R² value, compared to the relatively low R² obtained with cross-validation, may however confirm the model over-fitting and its poor transferability to new (“unknown”) contexts. Such an issue is common in regression models [78]. A complementary cross-validation, exploiting additional field data on the cells that were predicted and not used for the model training, could be carried out. Although RF is applicable when the number of variables is high, due to its potential for prediction, some studies highlighted the importance of selecting a limited number of predictors before making a prediction [79]. This was the purpose of selecting variables using MFA in this study. For future studies, other methods of variable selection could be tested to improve our results. However, the predictions appear coherent and are promising for further analysis. This kind of prediction could be directly used as an input (environmental charge capacity) of the mechanistic model based on the bio-ecology of *Aedes* mosquitoes to model population dynamics [65]. This would significantly objectivize the environmental charge capacity estimation which is, in practice, mainly estimated by expertise in order to implement such a mechanistic model. Unlike previous studies using positive breeding sites, the originality of this study lies in the direct use of potential breeding sites. In French Guiana, potential breeding sites data were used in research work for the first time, and the protocol must be adapted to the specific needs of this study in terms of predictive modeling. We expect that vector control will benefit from this research, helping update the design of entomological sampling to improve vector control strategies based on house prospections, by integrating knowledge about urban landscapes. The recent study by Rodriguez Conzalez et al. [57] confirms that combining Very High-Resolution (VHR) imagery and landscape metrics has the potential to improve vector control surveillance strategies, determining an optimal spatial repartition of ovitraps in complex urban environments. A similar approach could be carried out to improve sampling strategies in surveillance based on house prospection.

## Conclusion

We investigated the relationships between urban landscapes descriptors and potential breeding site distribution with a focus on the composition and three-dimensional structure of urban environments at a fine spatial scale, avoiding prior application of classification methods to better capture the complexity and heterogeneity of the landscape. The originality of this study is also to focus on the relevance of considering potential breeding sites for a more comprehensive understanding of the capacity of the territory to “produce” *Aedes* mosquitoes. We used a multifactorial analysis to explore relationships between potential breeding sites and urban landscape variables and select the most discriminatory variables. Such variable selection yielded improved performances in Random Forest models for predicting potential breeding sites. However, the challenge of predicting breeding site number as a function of their type persists, particularly due to the wide disparity in the numbers of breeding sites observed from one type of site to another. The study also highlighted the difficulty of identifying predictive variables relevant to all breeding site types. While texture variables alone did not exhibit sufficient performance, they proved valuable when combined with other variables extracted from remote sensing, showing the importance of both buildings and urban vegetation in explaining the distribution of potential breeding sites. The RF model achieved an excellent goodness of fit, but also overfitting, suggesting a difficulty in its generalization to new situations. This could be addressed by testing other cross-validation methods, finding better ways of filtering data (to minimize noise such as the errors in breeding site categorization and geolocalisation) and selecting predictors, and by enriching the learning set with data provided by different contexts. Although this research has limitations, it offers a first approach in the use of routinely collected data on breeding sites by vector control agencies in a research framework highlighting the need to include an objective remotely sensed characterization of the urban environment to improve vector control strategies.

## Electronic supplementary material

Below is the link to the electronic supplementary material.

Supplementary Material 1

Supplementary Material 2

Supplementary Material 3

Supplementary Material 4

Supplementary Material 5

Supplementary Material 6

Supplementary Material 7

Supplementary Material 8

## Data Availability

The urban datasets generated and analyzed, as well as the R codes developed during the current study, are available in the Forge IRD repository, https://forge.ird.fr/espace-dev/personnels/teillet/aedes_breeding_sites_modelling.git.

## Abbreviations

LULC: Land use and land cover
NDVI: Normalized difference vegetation index
NDWI: Normalized difference water index
VHR: Very High Resolution
CTG: Collectivité Territorial de Guyane
FT: Fourier Transform
PCA: Principal Component Analysis
CA: Total Class Area
CPLAND: Total class area percentage
LPI: Largest patch index
LSI: Landscape shape index
PD: Patch density
SHAPE_MN: Mean shape
CONTIG_MN: Mean of Contiguity
