# Supplementary material for: Exploring fine-scale urban landscapes using satellite data to predict the distribution of Aedes mosquito breeding sites
Source: Int J Health Geogr. 2024 Jul 7;23:18. doi: 10.1186/s12942-024-00378-3 (PMC11229250; doi:10.1186/s12942-024-00378-3)
Supplement: Supplementary file 1 — Supplementary Material 1 [file 12942_2024_378_MOESM1_ESM.pdf]

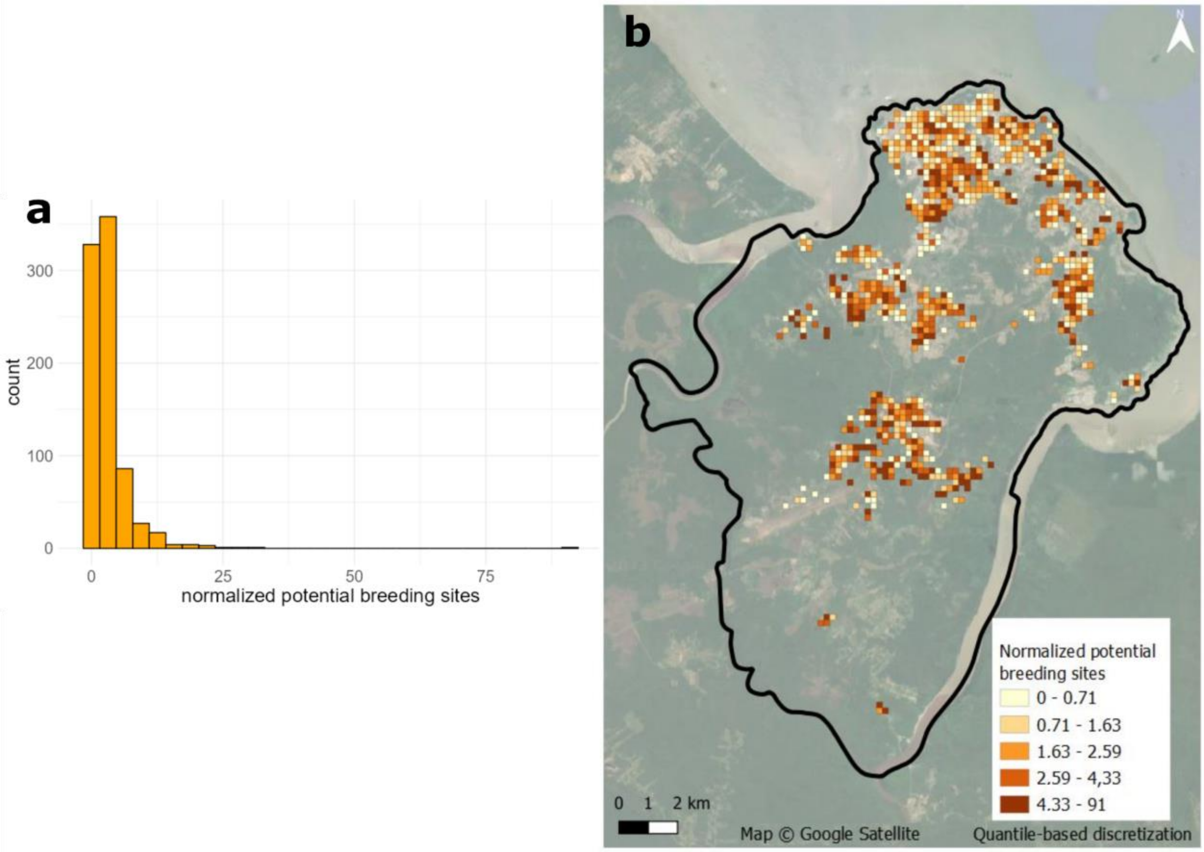

Additional file 1: (a) Count of normalized potential breeding sites per grid cell and (b) Normalized potential breeding sites per grid cell over Cayenne Island.
