# Supplementary material for: Exploring fine-scale urban landscapes using satellite data to predict the distribution of Aedes mosquito breeding sites
Source: Int J Health Geogr. 2024 Jul 7;23:18. doi: 10.1186/s12942-024-00378-3 (PMC11229250; doi:10.1186/s12942-024-00378-3)
Supplement: Supplementary file 3 — Supplementary Material 3 [file 12942_2024_378_MOESM3_ESM.pdf]

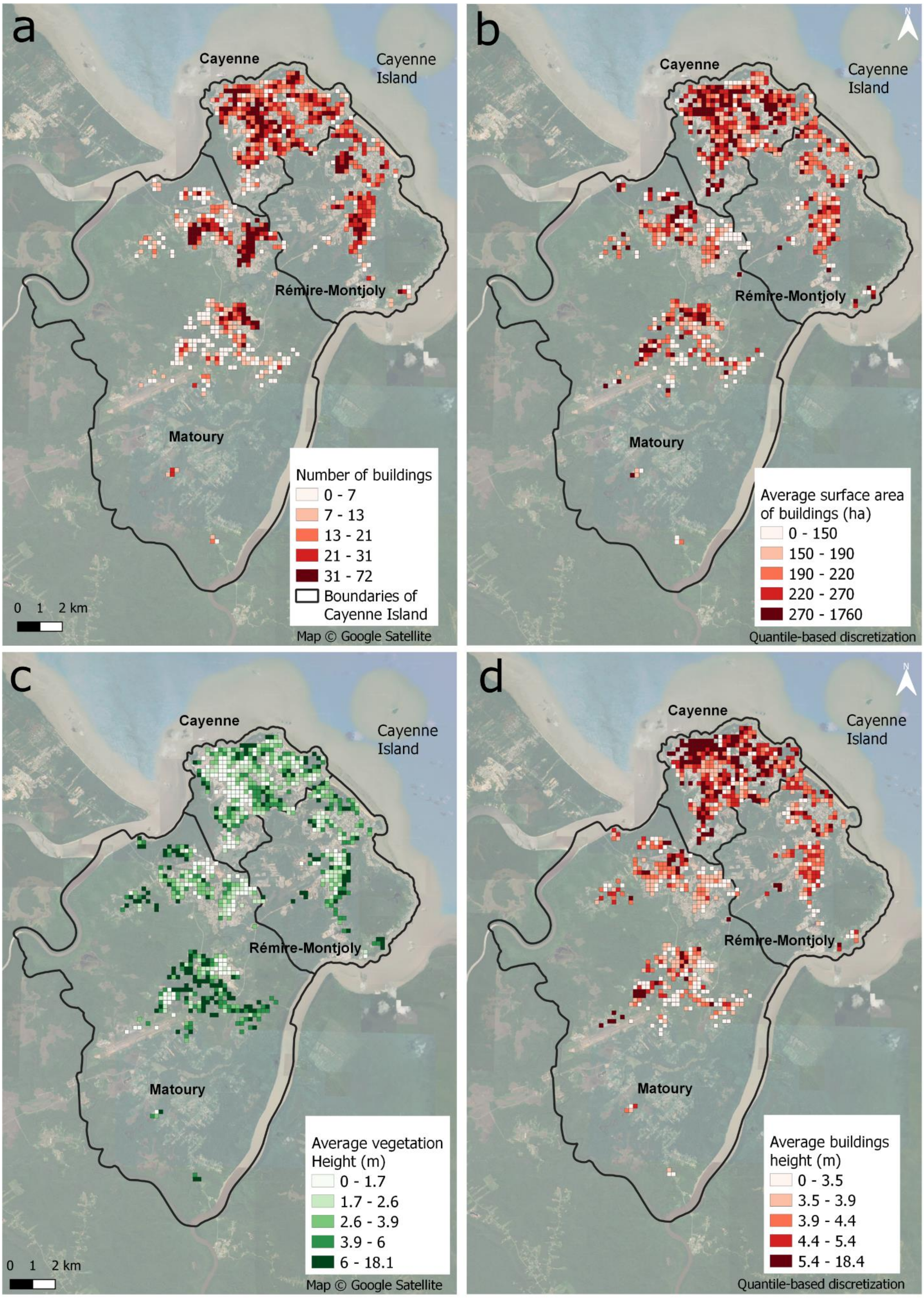
a
Cayenne
Cayenne
Island
Rémire-Montjoly
Matoury
Number of buildings
0 - 7
7 - 13
13 - 21
21 - 31
31 - 72
Boundaries of
Cayenne Island
0 1 2 km
Map © Google Satellite
b
Cayenne
Cayenne
Island
Rémire-Montjoly
Matoury
Average surface area
of buildings (ha)
0 - 150
150 - 190
190 - 220
220 - 270
270 - 1760
Quantile-based discretization
c
Cayenne
Cayenne
Island
Rémire-Montjoly
Matoury
Average vegetation
Height (m)
0 - 1.7
1.7 - 2.6
2.6 - 3.9
3.9 - 6
6 - 18.1
0 1 2 km
Map © Google Satellite
d
Cayenne
Cayenne
Island
Rémire-Montjoly
Matoury
Average buildings
height (m)
0 - 3.5
3.5 - 3.9
3.9 - 4.4
4.4 - 5.4
5.4 - 18.4
Quantile-based discretization

Additional file 3: building information and vegetation in Cayenne Island: (a) Number of buildings per grid cells ; (b) Average surface area of buildings (ha); *(c)* Average vegetation height; and (d) Average building height
