# Supplementary material for: Exploring fine-scale urban landscapes using satellite data to predict the distribution of Aedes mosquito breeding sites
Source: Int J Health Geogr. 2024 Jul 7;23:18. doi: 10.1186/s12942-024-00378-3 (PMC11229250; doi:10.1186/s12942-024-00378-3)
Supplement: Supplementary file 4 — Supplementary Material 4 [file 12942_2024_378_MOESM4_ESM.pdf]

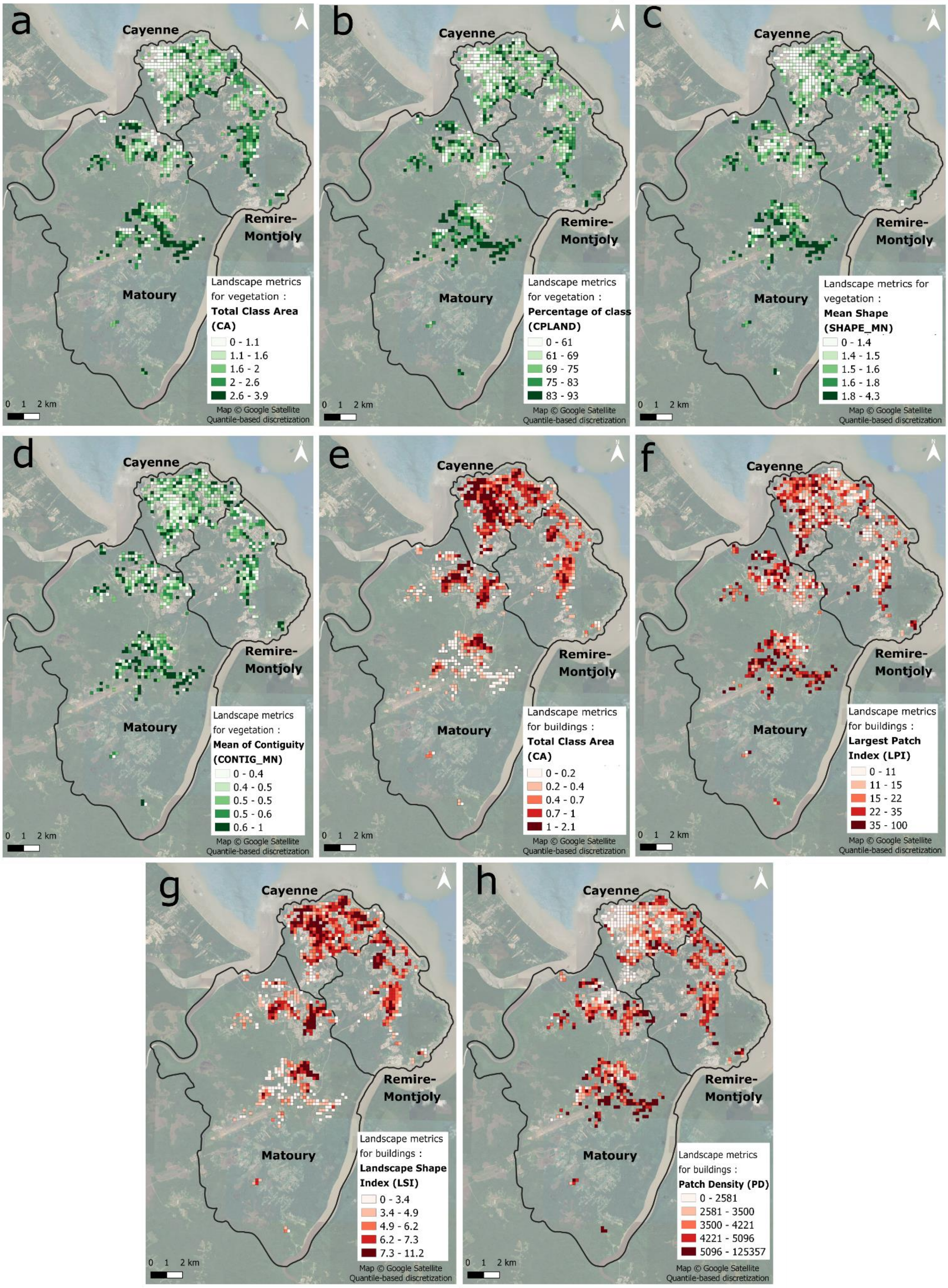

Additional file 4: Landscape metrics applied to vegetation: (a) Total Class area (CA); (b) Percentage of class (CPLAND); (c) Mean Shape (SHAPE_MN); (d) Mean of Contiguity (CONTIG_MN); Landscape metrics applied to buildings: (e) Total class area

(CA); (f) Largest Patch Index (LPI); (g) Landscape Shape Index (LSI); (h) Patch Density (PD).
