# Supplementary material for: Exploring fine-scale urban landscapes using satellite data to predict the distribution of Aedes mosquito breeding sites
Source: Int J Health Geogr. 2024 Jul 7;23:18. doi: 10.1186/s12942-024-00378-3 (PMC11229250; doi:10.1186/s12942-024-00378-3)
Supplement: Supplementary file 5 — Supplementary Material 5 [file 12942_2024_378_MOESM5_ESM.pdf]

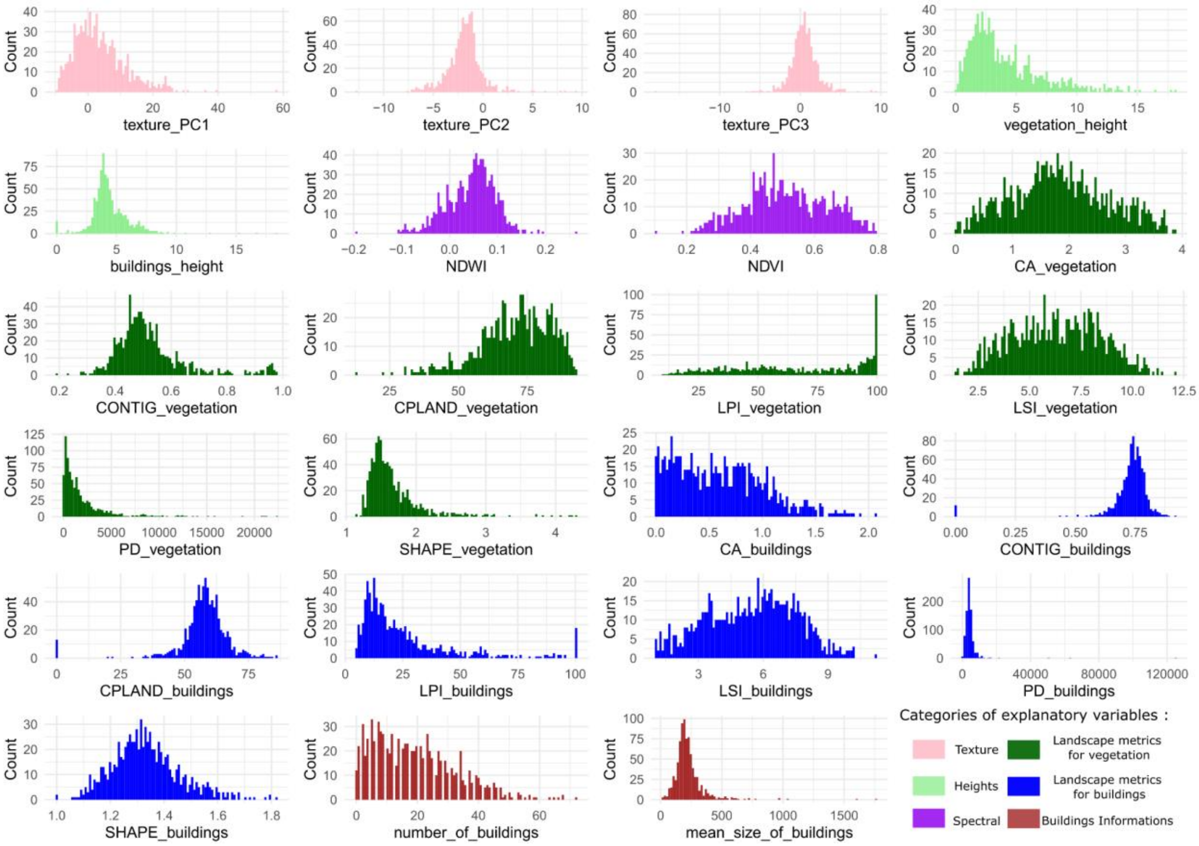

Additional file 5: Distribution of all urban landscapes explanatory variables (indicates variable categories tested in RF models). Largest Patch Index (LPI); Landscape Shape Index (LSI); Patch Index (PD); Percentage of class (CPLAND); Mean Shape (SHAPE); Mean of Contiguity (CONTIG).
