# Supplementary material for: Exploring fine-scale urban landscapes using satellite data to predict the distribution of Aedes mosquito breeding sites
Source: Int J Health Geogr. 2024 Jul 7;23:18. doi: 10.1186/s12942-024-00378-3 (PMC11229250; doi:10.1186/s12942-024-00378-3)
Supplement: Supplementary file 6 — Supplementary Material 6 [file 12942_2024_378_MOESM6_ESM.pdf]

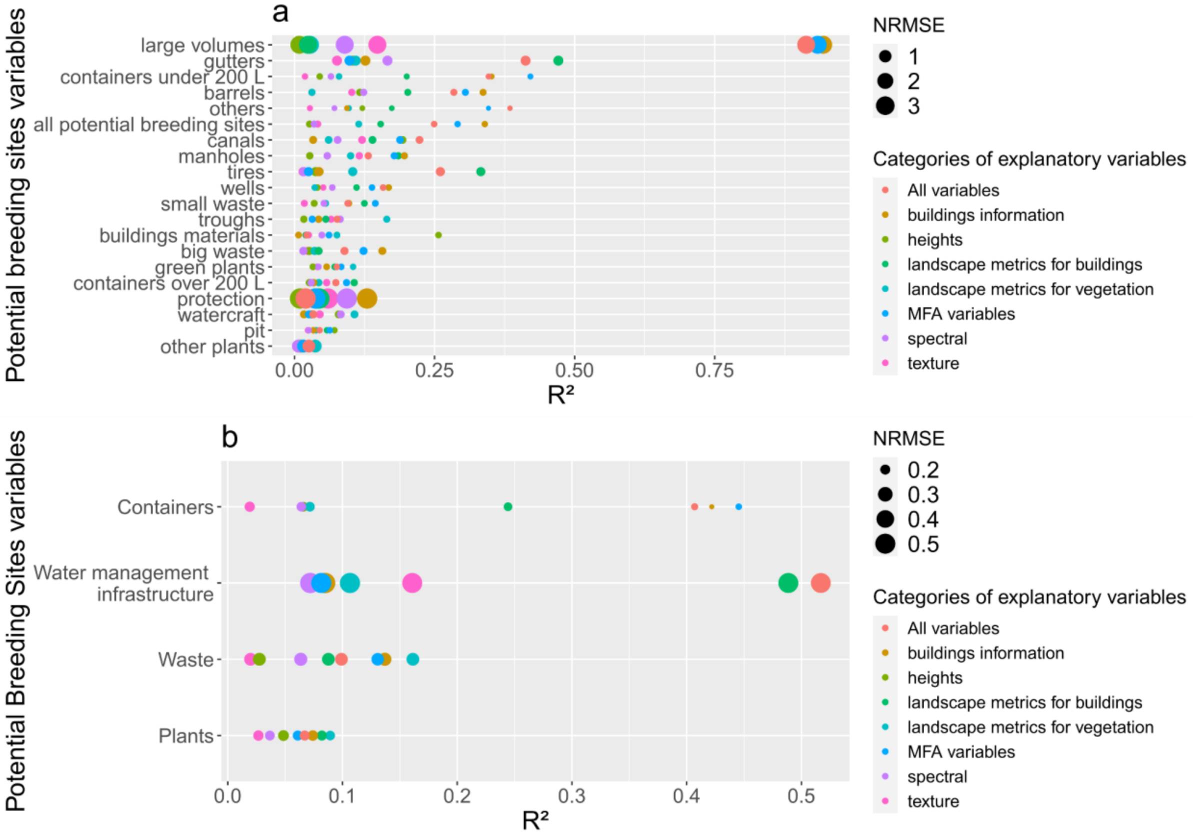

Additional file 6: RF models maximum R^2 and NRMSE values when considering different response variables and different groups of explanatory variables for (a) types of potential breeding sites, and (b) categories of potential breeding sites.
