# Supplementary material for: Exploring fine-scale urban landscapes using satellite data to predict the distribution of Aedes mosquito breeding sites
Source: Int J Health Geogr. 2024 Jul 7;23:18. doi: 10.1186/s12942-024-00378-3 (PMC11229250; doi:10.1186/s12942-024-00378-3)
Supplement: Supplementary file 7 — Supplementary Material 7 [file 12942_2024_378_MOESM7_ESM.pdf]

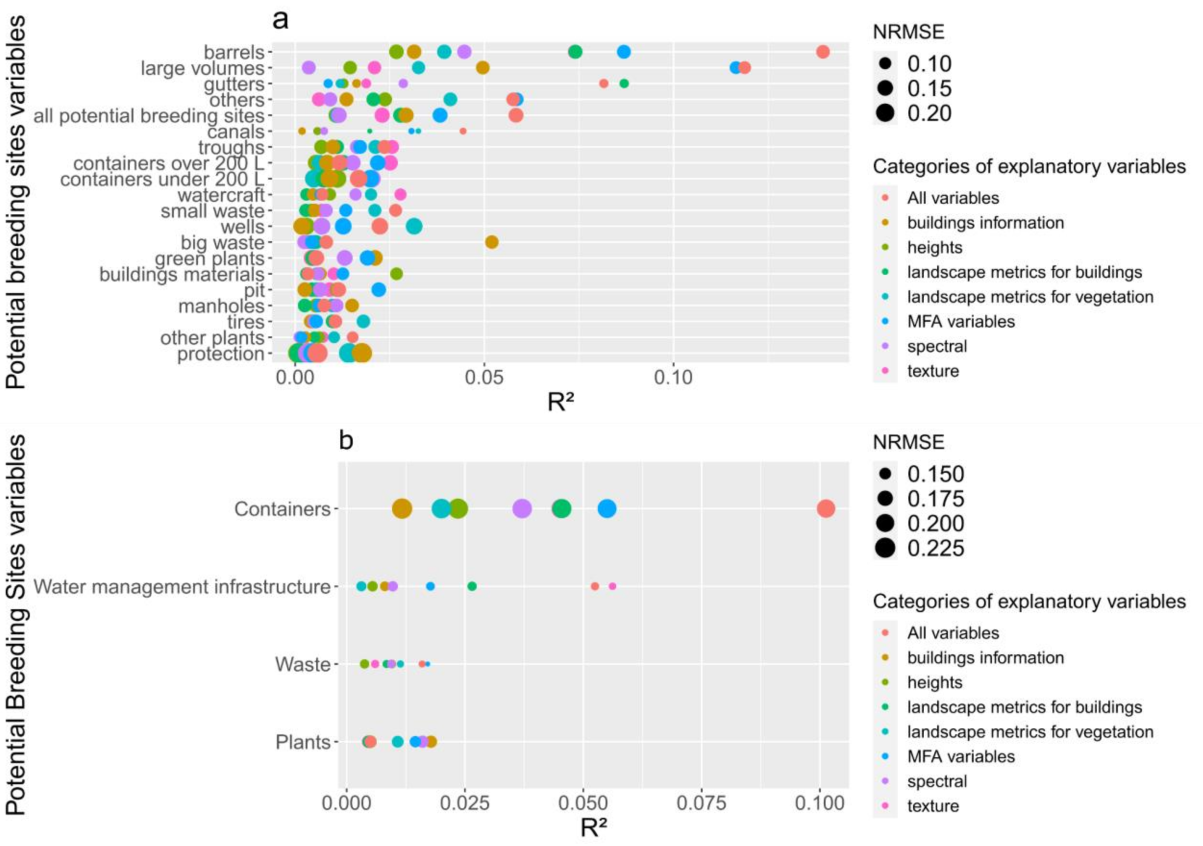

Additional file 7: RF models mean R^2 and NRMSE values when considering different response variables of normalized potential breeding sites and different groups of explanatory variables for (a) types of potential breeding sites, and (b) categories of potential breeding sites.
