# Supplementary material for: Exploring fine-scale urban landscapes using satellite data to predict the distribution of Aedes mosquito breeding sites
Source: Int J Health Geogr. 2024 Jul 7;23:18. doi: 10.1186/s12942-024-00378-3 (PMC11229250; doi:10.1186/s12942-024-00378-3)
Supplement: Supplementary file 8 — Supplementary Material 8 [file 12942_2024_378_MOESM8_ESM.pdf]

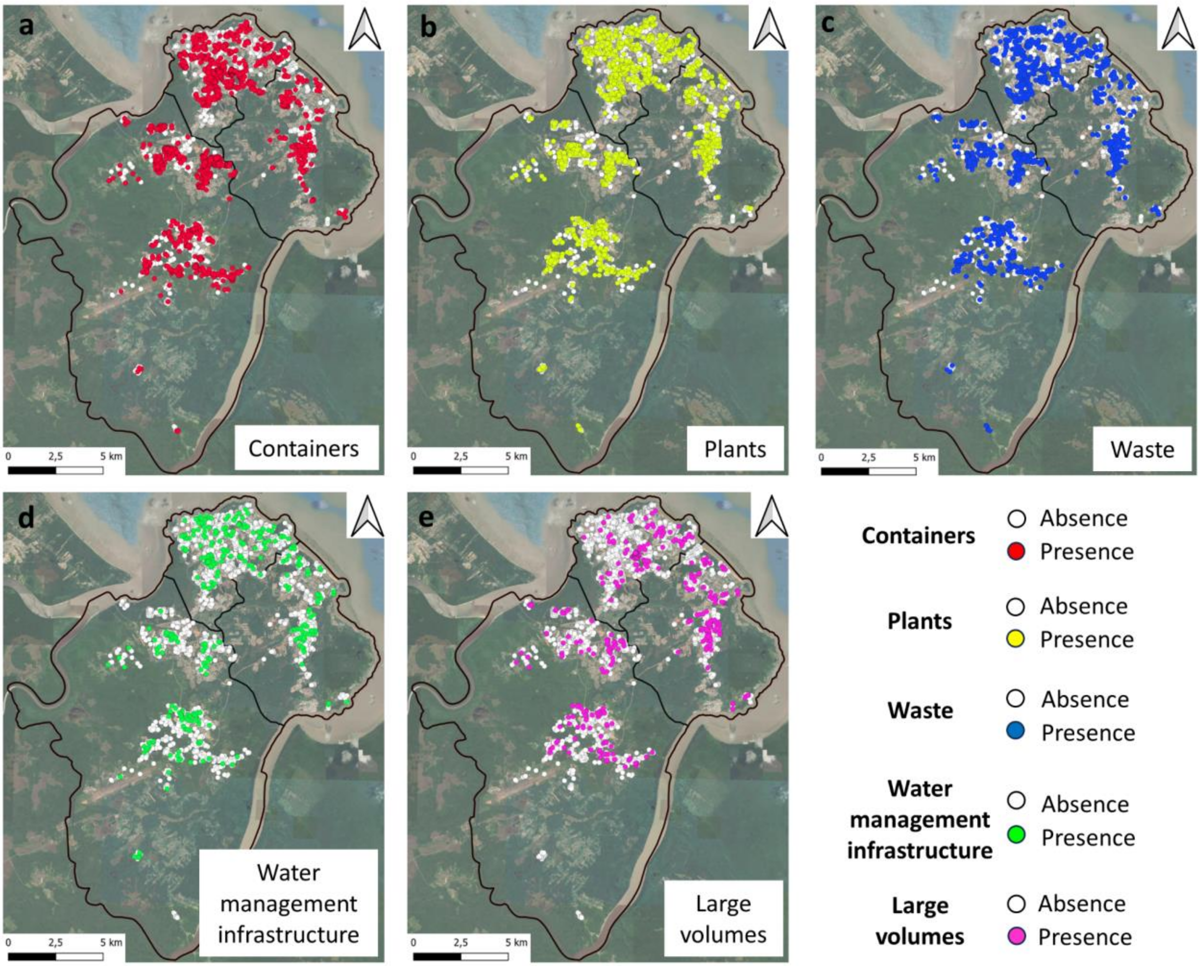

Additional file 8: Spatial distribution of potential breeding sites of each survey location for each category (a) Containers; (b) Plants; (c) Waste; (d) Water management infrastructure; (e) Large volumes.
